# Integrated Analyses of microRNAs Demonstrate Their Widespread Influence on Gene Expression in High-Grade Serous Ovarian Carcinoma

**DOI:** 10.1371/journal.pone.0034546

**Published:** 2012-03-29

**Authors:** Chad J. Creighton, Anadulce Hernandez-Herrera, Anders Jacobsen, Douglas A. Levine, Parminder Mankoo, Nikolaus Schultz, Ying Du, Yiqun Zhang, Erik Larsson, Robert Sheridan, Weimin Xiao, Paul T. Spellman, Gad Getz, David A. Wheeler, Charles M. Perou, Richard A. Gibbs, Chris Sander, D. Neil Hayes, Preethi H. Gunaratne, The Cancer Genome Atlas Research Network

**Affiliations:** 1 Dan L. Duncan Cancer Center, Baylor College of Medicine, Houston, Texas, United States of America; 2 Department of Medicine, Baylor College of Medicine, Houston, Texas, United States of America; 3 Gynecology Service, Department of Surgery, Memorial Sloan-Kettering Cancer Center, New York, New York, United States of America; 4 Computational Biology Center, Memorial Sloan-Kettering Cancer Center, New York, New York, United States of America; 5 Lineberger Comprehensive Cancer Center, University of North Carolina, Chapel Hill, North Carolina, United States of America; 6 Department of Internal Medicine, Division of Medical Oncology, University of North Carolina, Chapel Hill, North Carolina, United States of America; 7 Life Sciences Division, Lawrence Berkeley National Laboratory, Berkeley, California, United States of America; 8 Broad Institute of MIT and Harvard, Cambridge, Massachusetts, United States of America; 9 Department of Molecular and Human Genetics, Baylor College of Medicine, Houston, Texas, United States of America; 10 Department of Pathology, Baylor College of Medicine, Houston, Texas, United States of America; 11 Department of Biology & Biochemistry, University of Houston, Houston, Texas, United States of America; Harvard School of Public Health, United States of America

## Abstract

**Background:**

The Cancer Genome Atlas (TCGA) Network recently comprehensively catalogued the molecular aberrations in 487 high-grade serous ovarian cancers, with much remaining to be elucidated regarding the microRNAs (miRNAs). Here, using TCGA ovarian data, we surveyed the miRNAs, in the context of their predicted gene targets.

**Methods and Results:**

Integration of miRNA and gene patterns yielded evidence that proximal pairs of miRNAs are processed from polycistronic primary transcripts, and that intronic miRNAs and their host gene mRNAs derive from common transcripts. Patterns of miRNA expression revealed multiple tumor subtypes and a set of 34 miRNAs predictive of overall patient survival. In a global analysis, miRNA:mRNA pairs anti-correlated in expression across tumors showed a higher frequency of *in silico* predicted target sites in the mRNA 3′-untranslated region (with less frequency observed for coding sequence and 5′-untranslated regions). The miR-29 family and predicted target genes were among the most strongly anti-correlated miRNA:mRNA pairs; over-expression of miR-29a *in vitro* repressed several anti-correlated genes (including *DNMT3A and DNMT3B*) and substantially decreased ovarian cancer cell viability.

**Conclusions:**

This study establishes miRNAs as having a widespread impact on gene expression programs in ovarian cancer, further strengthening our understanding of miRNA biology as it applies to human cancer. As with gene transcripts, miRNAs exhibit high diversity reflecting the genomic heterogeneity within a clinically homogeneous disease population. Putative miRNA:mRNA interactions, as identified using integrative analysis, can be validated. TCGA data are a valuable resource for the identification of novel tumor suppressive miRNAs in ovarian as well as other cancers.

## Introduction

MicroRNAs (miRNAs or miRs) are ∼22 nt noncoding RNAs which target complementary gene transcripts for translational repression or mRNA cleavage [1]. Having been implicated in the initiation and progression of human cancers, miRNAs regulate processes such as cell growth, differentiation, and apoptosis [2]. A productive miRNA:mRNA interaction can occur with as little as six consecutive nucleotides, through pairing between the 5′-seed of the miRNA (located in nucleotides 2–7) and sequences which are largely localized in the 3′-untranslated regions (UTRs) of mRNA targets; consequently, a given miRNA can potentially impact hundreds of genes within and across diverse signaling pathways [3], [4], [5], [6], [7].

MiRNAs, along with gene copy number alterations and methylation of gene promoter regions, globally influence gene expression, which ultimately determines cellular behavior. The Cancer Genome Atlas (TCGA) project is a large-scale collaborative effort which seeks to comprehensively catalogue the molecular aberrations in various cancers. While the recent initial report from TCGA on 489 high-grade serous ovarian adenocarcinomas (487 of which had corresponding miRNA data) [8] presented a broad molecular picture of the disease, much in terms of miRNAs remains to be elucidated. Here, we comprehensively survey the miRNAs within the TCGA ovarian dataset, making use of the various molecular profile data types, miRNA and mRNA in particular, that have all been generated for the same set of tumors. Previous miRNA expression profiling studies of ovarian cancer have defined differentially expressed miRNAs in cancer relative to the corresponding normal control [9], [10], [11], [12], [13], [14], though in this present study, such a large dataset (n = 487 patients) allows us to more fully explore the diversity of miRNAs within a single ovarian cancer subtype.

Here, we present a number of findings on miRNAs in ovarian cancer, from various integration-based analyses. These analyses revolve around the basic question of whether the general rules of miRNA behavior, as we currently understand them, can be supported by corroborating patterns within human cancers. Our study serves to reinforce our current notions of basic miRNA biology, to demonstrate how current *in silico* miRNA-gene targeting predictions may be refined through integrative analysis, and to demonstrate the rich resource of TCGA in identifying miRNA candidates for functional targeting in cancer. Our study also provides second-level data mining results for molecular biologists to more deeply explore specific miRNA-associated pathways in ovarian cancer.

## Results

### MiRNAs are influenced by both copy number alteration and genomic location

**Figure 1:**
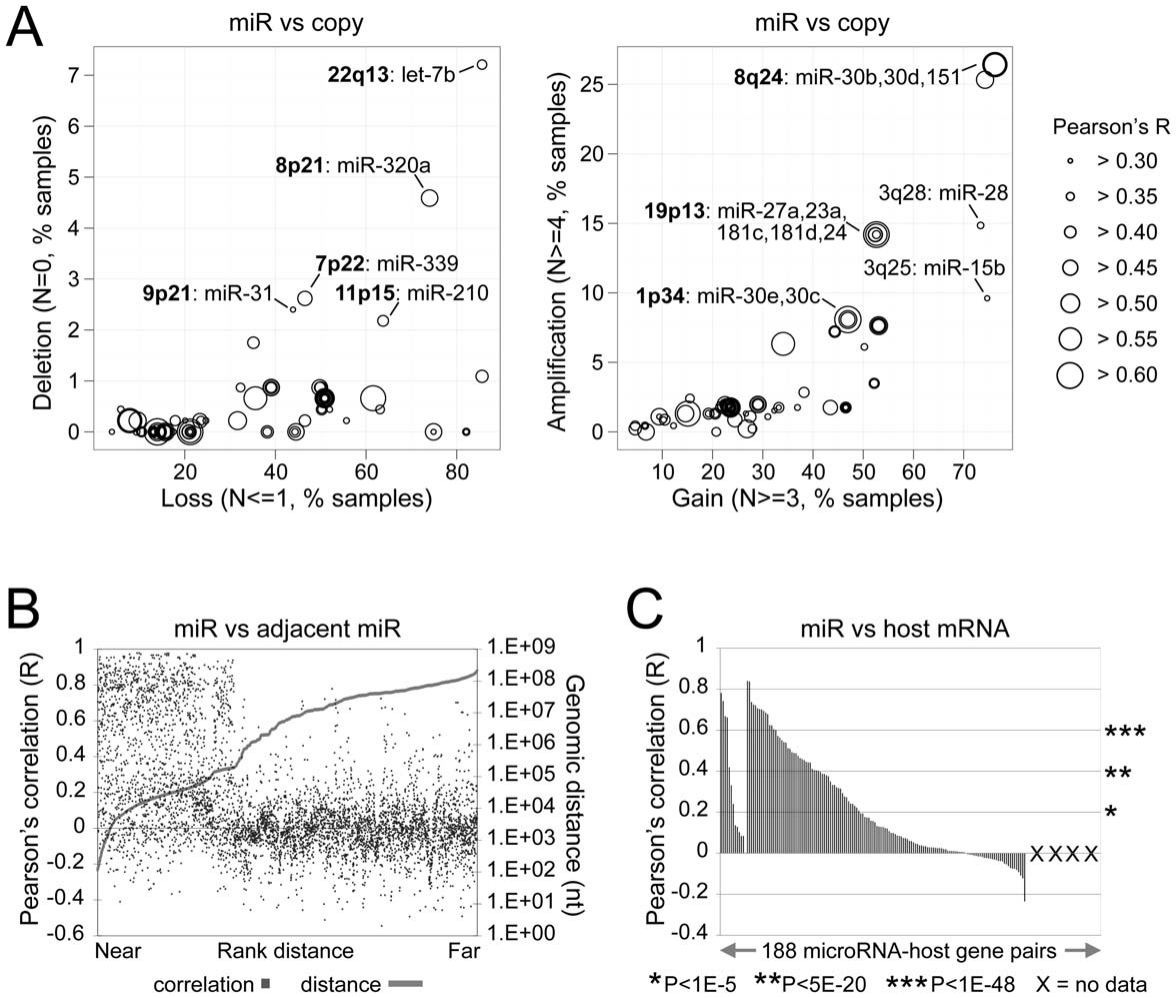
In ovarian tumors, expression patterns of miRNAs (miRs) are influenced by both copy number alteration (CNA) and genomic location. (**A**) MiRNAs correlated with CNA. Left plot shows hemizygous loss (N< = 1 copies) versus homozygous deletion (N = 0) for miRNA precursors. Correlation (Pearson's) between precursor DNA copy number and mature miRNA expression is indicated by symbol size, and only miRNAs with a minimum correlation (R>0.3) are included here; cytoband regions in bold represent focal CNA events. Similarly, right plot shows gain (N> = 3) versus amplification (N> = 4) for miRNAs having R>0.3 for precursor copy versus mature expression. (**B**) miRNAs are frequently coexpressed with neighboring miRNAs. Plot shows relationship between the distance separating miRNA loci and their coordinate expression. Each miRNA was paired with each of the others lying in the same orientation on the same chromosome. For each pair, the distance between the two loci (right axis) was ranked, and the correlation coefficient for their expression was plotted according this rank (left axis). (**C**) miRNAs are frequently coexpressed with host genes. For each of 188 miRNA-host gene pairs (same orientation), the correlation was computed both between miRNA and host gene expression; pair orderings are the same (“X”, no corresponding mRNA or gene copy data; p-values by two-sided t-statistic).

We examined the TCGA ovarian cancer datasets, representing 487 tumors profiled for miRNA expression, for patterns of correlation between the miRNAs and other molecular features, to see whether the overall trends observed would fit our initial expectations. To begin with, we considered that miRNAs with expression levels frequently altered by changes in DNA copy number may reveal a subset of miRNAs under clonal selection in the tumors; such miRNAs would be of potential interest as candidate oncomiRs or tumor suppressive miRs. We therefore systematically analyzed miRNAs for both loss and gain of DNA copy number associated with a concordant change in mature miRNA expression level (Figure 1A, Dataset S1). This analysis revealed several miRNAs in focally amplified and deleted genomic regions. In particular, let-7b was the most frequently deleted miRNA having both recurrent hemizygous genomic loss (86% of samples) and homozygous deletion (7.2%). Another deleted miRNA, miR-31, was recently found by our group to suppress ovarian cancer cell proliferation [10]. Four members of the miR-30 family were among the most frequently amplified miRNAs. Interestingly, these members were encoded at two different focally amplified loci (8q24 and 1p34) and all four miRNAs showed strong concordant change in mature miRNA expression.

In our data, we also found miRNAs to be frequently coexpressed with neighboring miRNAs as anticipated. Previously, when examining miRNA expression profiles in a small dataset of 24 normal human tissues, Baskerville and Bartel found evidence that proximal pairs of miRNAs are generally coexpressed (suggesting that they are processed from polycistronic primary transcripts), and that intronic miRNAs are usually coexpressed with their host gene mRNA (suggesting that they both derive from a common transcript) [15]. To extend these preliminary observations to ovarian cancer (thereby reinforcing current notions of miRNA biology as well as the integrity of our TCGA data), we made pairwise comparisons for each chromosome between the expression profiles of all miRNAs oriented in the same direction, calculating for each pair a correlation coefficient; the results showed that most miRNA genes within 50–100 kb of each other had highly correlated expression patterns (Figure 1B). Notably, at distances beyond 100 kb (exceeding the length of most human genes), the correlation between pairs dropped dramatically to zero. While DNA copy number alterations (CNA) undoubtedly influence gene and miRNA expression in cancer [10], [16], pairwise correlations in copy number levels between proximal miRNAs showed a very different pattern from the pairwise expression correlations; high proximal correlations for copy number extended for >1 Mb in length, with no dramatic drop (Figure S1A).

Approximately 177 of the 558 mature human miRNAs profiled are located in the genome within the introns of host genes, and we found miRNAs to be frequently coexpressed with these host genes in our data. For each of 188 miRNA-host gene pairs (each comprised of a miRNA located within the boundaries of a known gene, same orientation, where some mature miRNAs have multiple genomic locations), we computed the correlation between miRNA and host gene expression. MiRNA-host gene pairs tended to be strongly correlated with each other and, with 52% of the miRNA-host gene pairs with available data showing significant positive correlation (*P*<0.01), in agreement with previous studies [17], [18] (Figure 1C). As expected, miRNA expression was also correlated with host gene copy number, though the correlations were not as strong as for gene expression (Figure S1B).

### Diversity of miRNA and gene expression patterns suggestive of ovarian tumor subtypes

Previously, unsupervised clustering of miRNA expression data had suggested three general groupings or subtypes–with designations C1, C2, and C3–of high-grade serous ovarian tumors. The C1 subtype had been associated with worse patient survival as compared to the other two subtypes; in addition, the C1 subtype overlapped somewhat with the gene expression-based “proliferative” subtype, and the C2/C3 subtypes overlapped more with the gene-based “mesenchymal” subtype [8]. The average “silhouette” widths of the miRNA-based clusters had indicated them to be somewhat weakly defined with substantial within-group heterogeneity, while still reflecting patterns of biological diversity [8]. As described below, here we sought to further characterize these miRNA-based clusters, using our mRNA and miRNA expression data.

**Figure 2:**
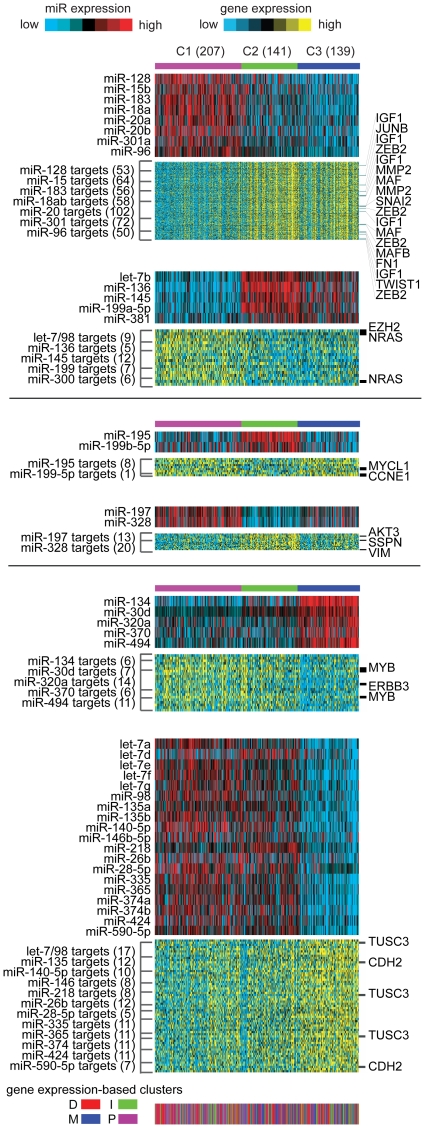
MiRNA correlates of molecular subtype, with associated gene expression patterns. Unsupervised clustering of miRNA expression data had identified three subtypes (C1–C3) of high-grade serous ovarian tumors. For miRNAs and genes differing significantly between the groups (t-test *P*<0.01, fold change>1.5, any subtype compared to the other tumors), predicted miRNA:mRNA functional pairs were defined, based on both anti-correlation in expression and predicted miRNA targeting interaction (both miRanda and TargetScan). For each miRNA:mRNA group (e.g. miRNA high/gene low in C1 versus other tumors), expression patterns are represented as heat maps (rows, miRNAs or gene transcripts; columns, profiled samples).

Given the role of miRNAs in repressing gene expression, we might anticipate that miRNAs and their mRNA targets in general would appear anti-correlated in expression in human tumors. A number of computational algorithms—the most well-known of these being miRanda [4] and TargetScan [7]—have been developed to predict the targeting of a given gene transcript by a specific miRNA (based on both sequence alignment of miRNA to gene 3′-UTR and on species conservation). By integration of both miRNA and gene expression patterns using previously-used approaches [10], we could define putative miRNA:mRNA functional pairs underlying the miRNA-based subtypes. Using the set of miRNAs and genes differing significantly between the groups, miRNA:mRNA pairs were defined by both predicted targeting association and anti-correlation in expression patterns (with the miRNA high and the gene low specifically in the given subtype, or vice versa) (Figure 2 and Dataset S2). MiRNAs high in C1 and C2 tumors included let-7 family members (excluding let-7b). Potential gene targets of interest included genes upregulated during epithelial-to-mesenchymal transition (EMT), such as *ZEB2*, *MMP2*, *SNAI2*, *FN1*, *TWIST1*, which were all high in the (mesenchymal-associated) C2 and C3 tumors; furthermore, C2 tumors had high vimentin mRNA and low E-cadherin mRNA.

### MiRNAs correlated in expression with worse patient outcome

**Figure 3:**
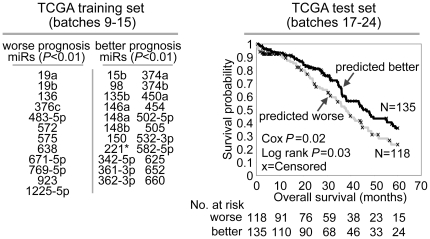
MiRNAs correlated with patient survival in ovarian cancer. Using a training dataset of TCGA miRNA expression profiles, a prognostic miRNA signature was defined (left panel) and then applied to a test dataset (right panel), each tumor being assigned a score measuring how well the tumor's expression patterns reflected those of the signature pattern. Kaplan-Meier analysis (log-rank tests) compares time to death for ovarian cancer patients showing higher risk (prognostic score>0) versus lower risk (prognostic score<0). Univariate Cox test treats the prognostic score as a continuous variable. (miR-923 was recently determined to be an rRNA fragment, according to miRBase.).

A set of molecular correlates of patient outcome is another resource TCGA data may provide to the research community. Previously, we identified a gene transcriptional signature predictive of overall survival in ovarian cancer [8]. Here, we carried out a similar analysis to define a miRNA signature of patient prognosis. In a training subset of 228 ovarian tumors (with outcome data, TCGA batches 9–15), 34 human miRNAs were individually correlated with time to death (Figure 3A, *P*<0.01, univariate Cox, average signal>50 units). Each of the 253 validation samples (batches 17–24) was assigned a prognostic score, reflecting the similarity between its expression profile and the prognostic miRNA signature pattern; the signature showed statistically significant associations with survival (Figure 3A, Log rank *P* = 0.03, miRNA risk index>0 vs <0, and *P* = 0.02, univariate Cox of the prognostic score as a continuous variable). Similar attempts at defining miRNA signatures of response to platinum therapy were unsuccessful (Document S1); however, as platinum-resistance is most likely a complex and multifactorial process, a role for miRNAs in platinum-resistance cannot be ruled out. A previous study [19] identified three miRNAs being associated with outcome in ovarian cancer; two of these miRNAs, miR-337 and miR-410, were also significant (*P*<0.05) in our training dataset. While a previous study had described an association between Dicer and Drosha expression levels and overall survival in ovarian cancer [20], we did not observe this association in our data (Figure S2).

Similar to what was done for the subtype-specific miRNAs, miRNA:mRNA predicted pairs were defined, using the larger set of miRNAs and genes correlated with outcome (*P*<0.05, univariate Cox) but in opposite directions to their predicted interactors (Figure S3 and Dataset S3). In the validation cohort, we compared the miRNA-based prognostic signature scores with the gene-based prognostic signature scores generated previously [8]. The correlation between the two sets of scores was statistically significant, though not high (Pearson's R = 0.2, *P*<0.001, Figure S4A). An additional set of scores obtained by averaging both miRNA and gene scores was significant by univariate Cox (*P* = 0.003), though a multivariate model combining the two scores gave indeterminate results, with each score trending towards significance (*P*< = 0.07 for each, Figure S4B). In a three-way analysis separating tumors with high or low scores (>0 or <0, respectively) for both gene and miRNA from the rest of the tumors, there was good separation between the groups (Log rank *P* = 0.01), with the mixed group (high for miRNA, low for gene, or vice versa) showing an intermediate outcome as compared to the predicted worse versus better groups (Figure S4C). In conclusion, miRNA expression patterns may complement gene expression patterns in predicting survival, with further study warranted; this issue of integrating miRNA and mRNA with survival data has also been examined elsewhere, using different analytical methods [21].

Some miRNAs correlated with disease progression could conceivably have a functional role in ovarian cancer; miRNAs correlated with better patient prognosis, for example, could be considered candidate tumor suppressors. MiR-148a, one of our better prognosis miRNAs, was recently found to inhibit proliferation in ovarian cancer cells [22]. With an aim towards uncovering new candidates for therapeutic targeting, we over-expressed miR-26b *in vitro*, which, while not our top significant miRNA, was correlated with better prognosis (*P*<0.05), as well as previously shown as inducing apoptosis in breast cancer [23]. Interestingly, miR-26b inhibited proliferation of HEYA8 but not OVCAR-8 cells (Figure S5, both cell lines being derived from ovarian cancers), though cell line-specific effects of miRNAs in ovarian cancer have been reported previously (possibly reflecting genotypic differences) [10]. One other miRNA from our signature that we tested, miR-146a, had no effect in either HEYA8 or OVCAR-8 (data not shown); no other miRNAs from this signature were tested *in vitro* by our group.

### MiRNAs and their predicted gene targets tend to be anti-correlated within ovarian tumors

**Figure 4:**
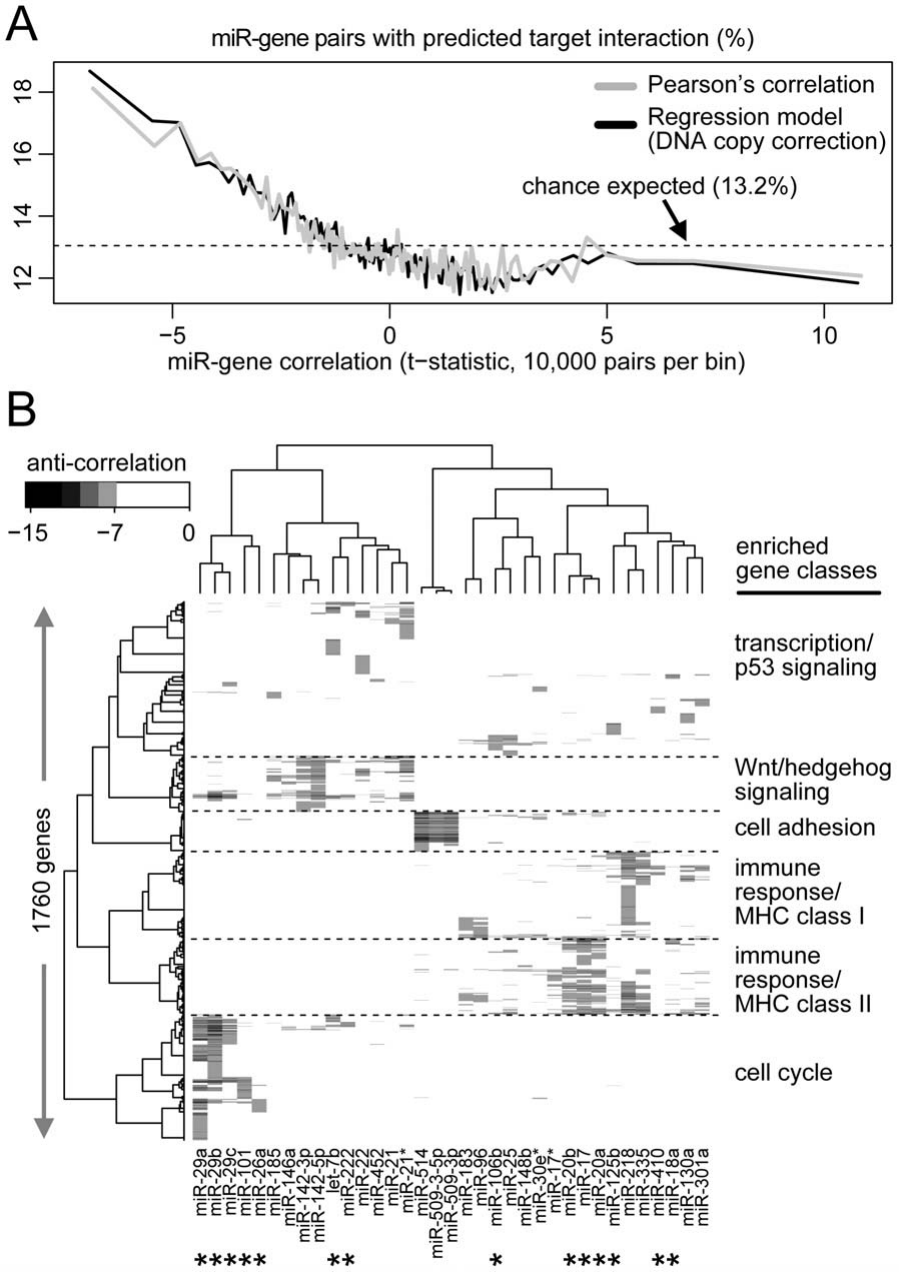
Correlations between miRNAs and genes in ovarian cancer. (**A**) MiRNAs and their predicted gene targets tend to be anti-correlated within ovarian tumors. Scatter plot showing mean correlation and fraction of predicted target interactions (miRanda-mirSVR score>0.1), using bins of 10000 miRNA:mRNA pairs (total number of pairs represented: 191 miRNAs X 8547 genes). Dashed line corresponds to chance expected baseline fraction (13.2%) of predicted target interactions. Correlations were computed using both Pearson's (gray line) and a simple linear regression model to account for ‘noise’ due to DNA copy alterations (black line). (**B**) Hierarchical clustering matrix (with Pearson's correlation coefficient as distance metric, Ward's Linkage) of correlation coefficients for all miRNA:mRNA pairs having a strong negative correlation (regression coefficient smaller than −7.0, only negative correlations represented). For each gene cluster, enriched gene classes are indicated. *, significant anti-enrichment (*P*<0.001, one-sided Spearman's rank, TargetScan or miRanda) for predicted targets within miRNA:mRNA correlations.

A key to studying miRNAs is identifying their gene targets. While miRNA targeting predictions made *in silico* (the vast majority being unvalidated) may have sizable rates of false positives and negatives, we hypothesized that considering correlations between gene and miRNA expression across a large panel of tumors could provide further support for potential miRNA:mRNA targeting relationships. To this end, we computed all possible miRNA:mRNA correlations across the 487 TCGA ovarian tumors, for the top expressed 191 miRNAs and 8547 genes. We then sorted the 191×8547 miRNA:mRNA pairs by low to high correlation, and found that among the most anti-correlated pairs, there was high enrichment for predicted miRNA:mRNA targeting interactions by miRanda algorithm (Figure 4A), where no such enrichment was observed for the positively correlated miRNAs:mRNAs. (This trend was observed when considering all other miRNAs and genes in addition to those most highly expressed, Figure S6.) In addition to validating the public target prediction databases as being enriched for true positives, this finding indicated that thousands of miRNA:mRNA targeting interactions are active in ovarian cancer and influence tumor gene expression heterogeneity.

The impact of CNA on expression level can vary greatly between genes, conceivably introducing bias when evaluating association of miRNA and gene expression levels. Therefore, in addition to a direct Pearson's correlation between miRNA and mRNA, we applied a simple linear regression model to account for ‘noise’ due to CNA, evaluating the association between expression levels of a miRNA and mRNA, when CNA status of the gene is held fixed. Interestingly, the Pearson's model and the regression model of miRNA:mRNA correlations both gave very similar overall results in terms of predicted target enrichment (Figure 4A), with the regression model's negatively correlated pairs showing slightly greater target enrichment (Figure S7). While, in general, CNA did not represent a major confounding factor, the regression model could identify individual miRNA:mRNA correlations which were missed by the Pearson's model, including miR-29a:*HARS2* (Figure S8).

As another way to globally represent miRNA:mRNA interactions in ovarian cancer, for all miRNA:mRNA pairs with the strongest negative correlation (regression coefficient <−7.0, based on the linear model), we clustered the matrix of correlation coefficients (Figure 4B, consisting of 1760 genes and 35 miRNAs, matrix data table available as Dataset S4), thereby grouping miRNAs when they are negatively correlated with same genes and vice versa. We then cut the gene dendrogram to extract 6 gene clusters (based on what appeared to be natural separations within the cluster tree), each of which was found to be uniquely enriched for different gene classes (Document S2), including a cluster with Wnt and Hedgehog pathway gene members, a cluster with cell adhesion genes, two clusters with immune response genes, and a cluster of cell cycle-related genes. For several individual miRNAs, the genes anti-correlated in expression were significantly enriched for *in silico* predicted targets (Figure 4B and Figure S9 and Figure S10).

### Genes anti-correlated with miRNAs are enriched for miRNA seeds predominantly in the 3′-UTRs

**Figure 5:**
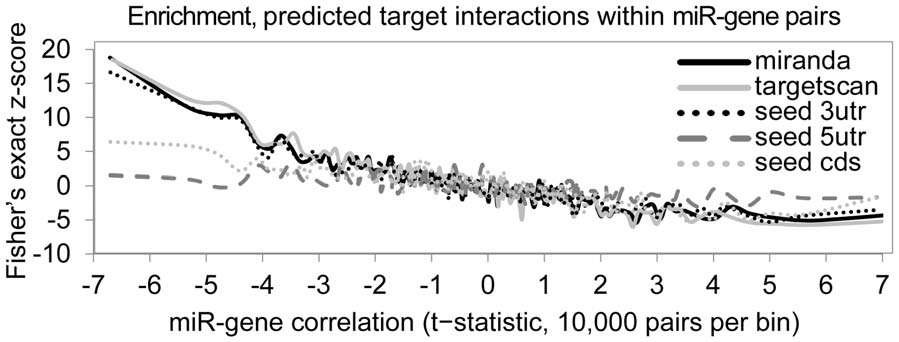
Gene transcripts with miRNA 7mer in the 3′-UTR tend to be anti-correlated with expression of the corresponding miRNA. Analogous to Figure 4A, scatter plot showing mean correlation versus significance of enrichment for predicted target interactions (enrichment expressed as a Fisher's exact z-score), when separately considering the following potential interactions: 7mer seed sequence in 3′-UTR (black dotted line), 7mer seed sequence in 5′-UTR (black dashed line), 7mer seed sequence in coding sequence region (“cds,” gray dotted line), miRanda prediction (black solid line), TargetScan prediction (gray solid line). Plot uses bins of 10000 miRNA:mRNA pairs (total number of pairs represented: 191 miRNAs X 8547 genes). Fisher's exact z-score of +/−2.57 corresponds to significant enrichment (nominal *P*<0.01) for predicted targets within miRNA:mRNA pairs.

Popular algorithms for miRNA targeting prediction, such as miRanda or TargetScan, rely on basic assumptions, including targeting within the gene 3′-UTR. While miRNAs are understood to typically bind 3′-UTRs, there have been a number of studies showing target sites in coding regions as being effective [24], [25], and others suggesting alternative 5′-UTR targeting [26], [27]. In order to determine the overall trends as indicated by our data, in terms of where miRNAs tend to bind, we examined miRNA:mRNA pairs anti-correlated in expression for the presence of miRNA seed sequences (7mers) in either the 3′-UTR, 5′-UTR, or coding sequence regions. Overall, high enrichment was found for 7mer seed sequences in the 3′-UTRs of genes anti-correlated with the corresponding miRNA (Figure 5), which was comparable to the enrichment patterns observed using miRanda or TargetScan predictions (which incorporate additional sequence features with seed sequence alignment); weaker enrichment patterns were found for seed sequences in coding regions, while no enrichment was evident for 5′-UTRs.

### MiR-29a impacts anti-correlated gene targets and ovarian cancer cell viability

**Figure 6:**
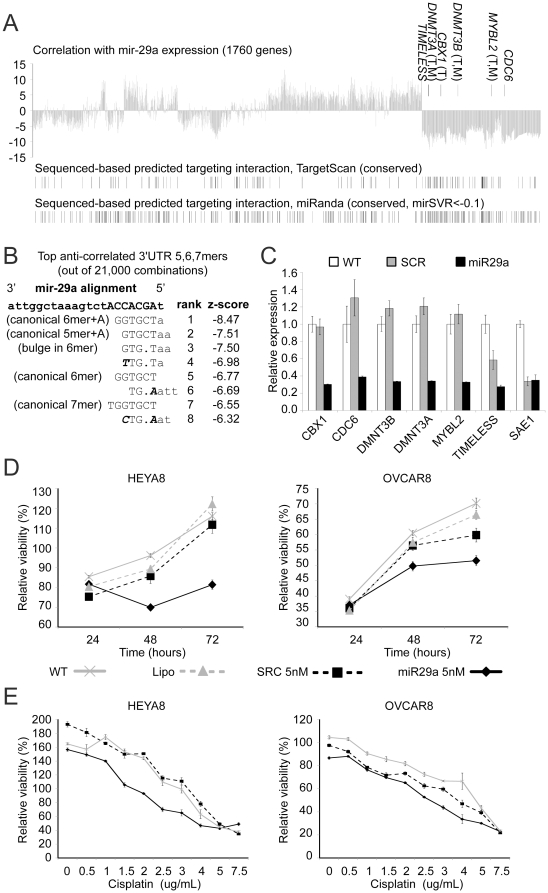
MiR-29a impacts anti-correlated gene targets and ovarian cancer cell viability. (**A**) Correlation of gene expression with miR-29a expression (genes from Figure 4B, same ordering). Predicted miR-29a targets are indicated. (**B**) Top eight words (of all 5, 6 and 7mers) enriched in 3′-UTRs of mRNAs anti-correlated with miR-29a expression (*FDR*<1e-6). (**C**) QPCR analysis showing relative quantity of selected miR-29a anti-correlated gene targets after miR-29a overexpression in HEYA8 ovarian cancer cells (SCR, scrambled control; WT, untreated; two-sided t-test *P*<0.05, mir-29a vs SCR and miR-29a vs WT, each comparison, except for *SAE1*). (**D**) MTS assays demonstrating the effect of miR-29a overexpression on proliferation of HEYA8 and OVCAR-8 cells (Lipo, lipofectamine-treated alone, no miRNA; two-sided t-test *P*<0.001, miR-29a vs each of three control groups at both 48 h and 72 h). (**E**) Effect of miR-29a (72 h) on proliferation, under a range of concentrations of cisplatin treatment (at 48 h). Error bars represent standard error.

The above results indicate widespread effects of miRNAs on gene expression in ovarian cancer, though any putative miRNA:mRNA interactions of interest remain to be validated. There are a number of ways one could arrive at candidate miRNAs for functional studies, using any of the results presented in our study. We focused our attention here on the miR-29 family, given its strong anti-correlation with many cell cycle-related genes (Figure 4B). Members of the miR-29 family have been demonstrated to act as tumor suppressors in acute myeloid leukemia and lung cancer, in part by reverting aberrant methylation patterns through its targeting of DNA methyltransferases (DNMT) and methylation-silenced tumor suppressors [28], [29]. Top anti-correlated genes of miR-29 in ovarian cancer included *DNMT3A* and *DNMT3B* (Figure 6A), suggesting a similar role for miR-29 in high-grade serous ovarian cancer. MiR-29a was under-expressed and *DNMT3A* mRNA was over-expressed in the DNA methylation subtype “MC2” (Figure S11); furthermore, possible targets of *DNMT3A* methylation in the ovarian tumors (having DNA methylation levels correlated with *DNMT3A* expression) were enriched for genes showing an impact by methylation (Figure S12, Table S1).

Genes anti-correlated with miR-29a were enriched for miR-29a targets as predicted by sequence analysis (either TargetScan or miRanda, Figure 6A). However, many *in silico* predicted targets did not show the anticipated anti-correlation patterns, again suggesting that by factoring in expression data, we could reduce the false positive rate for target predictions. Furthermore, as additional evidence for miR-29 activity, a correlation-based sequence motif analysis found that the miR-29 seed sequence complement was the top enriched motif in 3′-UTRs of mRNAs anti-correlated with miR-29a expression (Figure 6B), further suggesting that miR-29 directly regulates expression levels of many target mRNAs in the tumors; this analysis also showed strong enrichment for non-canonical miR-29a seed motifs (i.e. motifs not following the typical pattern of nucleotides 2–7) with a bulge in position 3 of the miR-29a sequence, suggesting that target prediction methods requiring perfect base pairing in the seed region of the miRNA target duplex could miss a substantial fraction of functional miRNA target interactions.

By forcing miR-29a expression *in vitro* in the ovarian cancer cell line HEYA8, we confirmed that a number of the genes anti-correlated with miR-29a—*DNMT3A*, *DNMT3B*, *CDC6*, *CBX1*, *MYBL2*, and *TIMELESS* (four of which were predicted direct targets)–were repressed by miR-29a (Figure 6C), which demonstrated these gene targets as relevant in both the *in vitro* functional models as well as the human tumor specimens; one gene tested, *SAE1*, showed anti-correlations but no functional repression. While miR-29 expression was not associated with survival (P>0.05, univariate Cox), forced miR-29a expression impacted cell proliferation in OvCar-8 and HEYA8 cell lines (Figure 6D) and had an additional effect on chemotherapeutic agent cisplatin in inhibiting the growth of these lines (Figure 6E).

## Discussion

Molecular profiling of cancers is thought to potentially provide important new classifications of patients and insight into observed heterogeneity within a given disease. Over ten years ago, global gene expression analyses of breast cancer defined distinct molecular subtypes, which encompassed much of the known heterogeneity of the disease in terms of histology [30]. Recently, gene expression profiles have sub-classified cancers such as glioblastoma and high-grade serous ovarian cancer [8], [31]; diseases which might be considered relatively homogeneous from a clinical standpoint actually show highly diverse patterns at the gene transcript level. In our study, we see extensive diversity of miRNA patterns within high grade serous ovarian cancer, suggestive of disease subtypes and patient outcome differences. We might expect these diverse miRNA patterns to be reflected in the gene expression data. Here, correlative analyses between mRNAs and miRNAs helped establish aspects of normal miRNA biology, such as the influence of host gene expression on intronic miRNAs, as being maintained in cancer.

Before we began our study, it was unclear whether a broad analysis of miRNAs would show them to have a widespread impact on gene expression programs in ovarian cancer, as we have now established through the clear patterns of anti-correlation observed between miRNAs and predicted targets. Numerous functional miRNA-gene targeting relationships have been validated in previous studies, using cell lines, and artificially modulating certain miRNAs *in vitro* can show clear effects on cell behavior. However, an overarching question with regards to cell line studies is whether these are truly relevant to human cancers. While data from experimental models (such as cell lines) help to establish cause-and-effect relationships in the laboratory, data from human tissues (such as cancer) can establish correlative (though not necessarily causal) relationships that arguably appear relevant to human disease; the combination of experimental and human tissue data therefore ought to provide the strongest support for the disease-specific relevance of a particular miRNA and its gene targets.

The integration of miRNA and gene expression data within the same large panel of tumors allows us to define miRNA:mRNA correlations that are indicative of miRNA targeting. The observed enrichment of *in silico* predicted miRNA targets within anti-correlated miRNA:mRNA pairs both helps strengthen our confidence in the *in silico* predictions (as based on the canonical rules of miRNA:mRNA interaction), as well as allowing us to prioritize those predictions that appear most relevant in ovarian cancer. We should note, however, that our overall findings are broad, allowing individual exceptions to the general rule (e.g. 5′-UTR targeting or miRNA-mediated upregulation of genes), and that our study does not rule out future discoveries regarding new rules of miRNA behavior not covered by our basic assumptions; furthermore, a whole gene array analysis is perhaps limited in being unable to detect alternative transcripts of the same gene differing in 3′-UTRs. Notwithstanding its limitations, our analytical approach allowed us to identify miR-29a, previously showing tumor-suppressive effects in other cancers, as having a similar role in ovarian cancer. More functionally-relevant miRNAs and their targets remain to be identified and explored, and TCGA data will remain a valuable resource for miRNA:mRNA integrated approaches to discovering novel candidate targets for cancer therapy in ovarian as well as other cancers.

## Materials and Methods

### Molecular profiling datasets

The set of 487 tumors analyzed were from the original TCGA set of 489 [8] (samples TCGA-04-1536 and TCGA-61-1911 did not have quality miRNA data at the time of this study). The miRNA array normalization steps are as follows. The gMeanSignal from raw array files (“level 1”) were quantile normalized and log transformed, removing duplicate samples and control probes (“level 2”). Multiple median centering steps set the median of every batch to the median of all batches: in brief, within each batch, we first subtracted the median for each miRNA, then calculated the across batch median and added it back to all samples within that batch; the resulting data were collapsed to miRNA levels (“level 3”). The level 3 miRNA data are available at the TCGA Data Portal [32]. For gene expression analysis, we relied on the previously described “unified” dataset [8].

### miRNA:mRNA correlation analysis

Differentially expressed genes and miRNAs were identified using two-sided t-test on log-transformed data. Java TreeView [33] represented expression patterns as color maps. MiRNA:mRNA targeting relationships for both subtype correlates and patient survival correlates were identified using TargetScan Human (release 5.0) [7] and miRanda (September 2008) [34]; SigTerms facilitated retrieval of putative miRNA:mRNA pairs [35]. Predicted targeting relationships for miRNA:mRNA correlations were identified using miRanda (microRNA.org, August 2010, conserved set). For estimating absolute miRNA precursor copy levels (Figure 1A), we used the cBio Cancer Genomics Portal [36]. The top 191 miRNAs X 8547 genes (Figures 4 and 5) were defined as: for miRNAs, those in the top 100 with highest signal in at least ten individual samples; for genes, with expression above the tumor sample median in at least ten samples. Using a previously-described statistical framework evaluating correlation of 3′-UTR oligonucelotide (word) occurrences and mRNA expression changes [37], we analyzed motifs enriched in 3′-UTRs of mRNAs anti-correlated with miR-29a expression, computing word association z-score with P-value for all 21,504 words of length 5–7.

### Survival analysis

The definition and validation of a prognostic miRNA signature was carried out essentially as described for the previously-defined prognostic mRNA (gene) signature [8], using the previously-defined training and validation subsets with expression values normalized within each subset to standard deviations from the median. Given the miRNA signature from the training dataset, the prognostic t-score was defined for each validation profile as the two-sided t-statistic comparing, within each tumor profile, the average of the poor prognosis miRNAs with the average of the good prognosis miRNAs.

### Cell cultures

OVCAR-8 cells were obtained from the NCI-Frederick Cancer DCTD Tumor/Cell Line Repository (Frederick, MD), and HEY-A8 cells were obtained from Gordon Mills (M.D. Anderson), both cell lines having been properly authenticated by their respective sources; cells were passaged in our laboratory for no more than two months after resuscitation. Cells were cultured in RPMI 1640 (Gibco) with 10% heat-inactivated fetal bovine serum (Denville Scientific) and penicillin-streptomycin (Invitrogen).

### miR-29a overexpression

Cell lines were transfected in 6-well plates (6×10^4^ cells/well) using 2.5 µl/well of Lipofectamine 2000 Transfection Reagent (Invitrogen) and 10 pmol of hsa-miR29a (Ambion), according to manufacturer's instructions. Control groups of cells were treated with transfection reagent alone (mock transfection), or transfected with pre-miR negative control #1 (Ambion).

### Proliferation assays

Cell were seeded (3000/well) in a 96 well plate and transfected using 0.1 µl/well of Lipofectamine 2000 Transfection Reagent (Invitrogen) and 0.5 pmol of hsa-miR29a (Ambion), with lipofectamine alone or pre-miR negative control #1 (Ambion) as controls. Cell proliferation was assayed at 24, 48, and 72 hrs post-transfection using the MTS-based CellTiter 96 cell proliferation assay (Promega, Madison, WI). The time course MTS assay experiments were run three times (separate days), each with a different set of biological quadruplicates (n = 12 per group); within each experiment run, the viability measures within each time point were centered on the mean of the WT group for the first run. For cisplatin treatment, cells were transfected as described above, and media was replaced after 24 hrs with media containing Cisplatin (Sigma) (0–7.5 µg/mL); viability was assayed 72 hrs post-transfection; experiments were run three times, each with a different biological replicate (n = 3 per group); for each run, viability measures within each concentration point were centered on the mean of values for the first run.

### Quantitative real-time PCR (QPCR)

Total RNA (60 ng) was reverse transcribed in a 40 µl reaction using the TaqMan® MicroRNA Reverse Transcription Kit (ABI). Custom primer sequences are in Table S2. QPCR was performed on a StepOne Real-Time PCR System (ABI) using Power- SYBR Green PCR Master Mix (ABI) in a 20 µl reaction and human ribosomal RNA 18 s as an endogenous control (which was itself not miR-29a-regulated, data not shown). The QPCR experiments were run four times (separate days), each with independent biological samples (n = 4 per group); within each experiment run, relative expression values were normalized to standard deviations from the mean.

## Supporting Information

Dataset S1 **miRNAs most correlated in expression with DNA copy number.**
(XLSX)

Dataset S2 **miR:mRNA pairs correlated with tumor subtype.**
(XLSX)

Dataset S3 **miR:mRNA pairs correlated with patient outcome.**
(XLSX)

Dataset S4 **miR:mRNA correlation matrix for top expression miRs and mRNAs.**
(XLSX)

Document S1 **Lack of correlation of miRNAs with platinum response.**
(DOC)

Document S2 **Functional gene classes associated with negatively correlation miR:gene pairs.**
(PDF)

Figure S1 **The observed patterns of miRNAs (miRs) being frequently coexpressed with neighboring miRNAs and host genes are not solely due to copy number alterations.** (A) As in main Figure 1A, but with the correlations between miRNA copy levels (SNP 1 M dataset, collapsed into miRNAs). (B) For purposes of comparison with the results of main Figure 1B, for each of 188 miRNA-host gene pairs (same orientation), the correlation was computed between miRNA expression and host gene copy (part D, using MSKCC 1 M CGH dataset, averaged by gene); ordering of mRNA-host gene pairs is the same between main Figure 1B and Supplemental Figure 1B; “X”, no corresponding mRNA or gene copy data.
(PNG)

Figure S2 **Lack of association between Dicer and Drosha levels with overall survival in the TCGA cohort.** A previous study of 111 invasive epithelial ovarian cancer samples found that the distribution of Dicer mRNA levels was bimodal, and that both Dicer and Drosha levels were positively associated with overall survival (Merritt et al, 2008). (A) Dicer mRNA was not bimodal in TCGA. (B) The data did not support a significant difference in overall survival between patients with low and high Drosha or Dicer levels. This was the case when comparing the top and bottom 50% of patients as well as the lower and upper quartiles (P>0.45, logrank test). A marginal improvement in overall survival was seen in patients where Dicer levels were in the extreme upper and lower quartiles (P = 0.026, logrank test), although median survival for uncensored patients was not improved in this case. Likewise, no association was revealed by Cox proportional hazards regression (P = 0.45 and P = 0.53 for Dicer and Drosha, respectively).
(PNG)

Figure S3 **miRNAs and associated genes correlated with patient survival in ovarian cancer.** (A) Using a training dataset of TCGA microRNA expression profiles, a prognostic gene signature was defined (left panel) and then applied to a test dataset (right panel), each tumor being assigned a score measuring how well the tumor's expression patterns reflected those of the signature pattern. Kaplan-Meier analysis (log-rank tests) compares time to death for ovarian cancer patients showing higher risk (prognostic score>0) versus lower risk (prognositc score<0). Univariate Cox test treats the prognostic score as a continuous variable. (B) As for part A, but using gene expression profiles to define survival gene correlates. (C) Numbers of predicted miRNA-mRNA functional pairs for each algorithm and intersection of algorithms based on anti-correlated expression in ovarian cancer. (D) Top enriched miRNA targeting associations (one-sided Fisher's exact of P<0.05) for the genes correlated with better prognosis, for the given algorithm.
(PNG)

Figure S4 **Comparison of prognostic signature scores derived from miRNA versus gene expression.** Previously (TCGA consortium, 2011), a gene expression signature of prognosis was derived and applied to the same validation dataset used to validate our miRNA prognostic signature. (A) Scatterplot comparing miR and gene prognostic scores in the validation cohort (N = 253). R-value and P-value by Pearson's. (B) Cox survival analysis of miR and gene prognostic signature scores (evaluated as a continuous variable). The scores were evaluated individually by univariate Cox, as well as an averaging of the two scores. By multivariate Cox, the scores were evaluated in the same model. (C) Kaplan-Meier analysis evaluating survival time for three groups of patients: score>0 for both miR and gene (pink), score<0 for both miR and gene (yellow), and all others (blue).
(PNG)

Figure S5 **miR-26b, associated with longer survival in ovarian cancer patients, impacts cell viability** ***in vitro*** **in HEYA8 cell line but not in OVCAR8.** (A) Kaplan-Meier analysis evaluating survival time for patient with higher versus lower levels of miR-26b. Univariate Cox test evaluates miR-26b expression as a continuous variable; Log rank test compares the top 25% of expressors with the rest of the patients. (B) MTS assays demonstrating the effect of miR-26b overexpression on proliferation of HEYA8 cells (Lipo, lipofectamine-treated alone, no miRNA; two-sided t-test P< = 0.01, miR-26b vs each of three control groups at both 48 h and 72 h). (C) No effect was observed for miR-26b on proliferation of OVCAR-8 cells. Over-expression and MTS experiments for miR-26b were carried out in the same manner as for the miR-29a experiments of Figure 6D.
(PNG)

Figure S6 **MiRNAs and their predicted gene targets tend to be anti-correlated within ovarian tumors.** Similar to main Figure 4A, except here all miRNAs and genes represented in the dataset are considered (A), in addition to the top most expressed miRNAs and genes (B), from Figure 4A, as well as the remaining miRNAs and genes expressed below the set threshold (C). miRNA:gene pairs are subdivided into bins of 10,000 each. Miranda predictions and Regression model results were used.
(PNG)

Figure S7 **MiRNAs and their predicted gene targets tend to be anti-correlated within ovarian tumors, with the regression model's negatively correlated pairs showing slightly greater target enrichment as compared to the Pearson's model.** Scatter plot showing *cumulative* mean correlation and fraction of predicted target interactions (miRanda-mirSVR score>0.1), using bins of 10,000 miRNA:gene pairs (total number of pairs represented: 191 miRNAs X 8547 genes). (Similar to main Figure 4A, except number of interactions is cumulated from low to high correlation.)
(PNG)

Figure S8 **miR-29a:HARS2 as an example demonstrating correction of CNA bias in miRNA:gene expression correlation.** Correlations were computed using both Pearson's correlation (scatter plot outlined in red) and a simple linear regression model to account for ‘noise’ due to DNA copy alterations (scatter plots outlined in blue).
(PNG)

Figure S9 **Within ovarian tumors, several specific miRNAs tend to be anti-correlated with their predicted gene targets.** Left panel shows hierarchical clustering matrix (with pearson correlation coefficient as distance metric,Ward's Linkage) of correlation coefficients for all miRNA:gene pairs (yellow = positive correlation; blue = negative correlation). The two right panels show the corresponding predicted targeting interaction (both PicTar and TargetScan algorithms) for the miRNAs/genes (same gene ordering). *, significant anti-enrichment (P<0.001, Spearman's rank, one-sided) for predicted targets within miRNA-to-gene correlations.
(PNG)

Figure S10 **For each miRNA in the miRNA:gene correlation matrix (from** **Figure 4** **), enrichment of predicted target genes within each of the six different gene clusters.** Enrichment (bottom panel) is given by the fraction of predicted target genes in a given cluster (miRanda miRSVR score<−1.0), divided by the background expected ratio (overall fraction of miRNA target genes among all genes measured on array and with predicted target sites of any human miRNA).
(PNG)

Figure S11 **Methylation subtype 2 is associated with combined low miR-29 and high DNMT3A levels.**
(PNG)

Figure S12 **Possible targets of DNMT3A methylation in the ovarian tumors (having DNA methylation levels positively correlated with DNMT3A expression level) show a significantly higher proportion of genes with mRNA expression levels strongly affected by DNA methylation (P<1.4Ee-26, Wilcoxon Rank-sum).**
(PNG)

Table S1 **DNA methylation probes most correlated with DMN3TA expression.**
(XLSX)

Table S2 **PCR primer sequences.**
(DOCX)
