# Supplementary material for: Integrated Analyses of microRNAs Demonstrate Their Widespread Influence on Gene Expression in High-Grade Serous Ovarian Carcinoma
Source: PLoS One. 2012 Mar 29;7(3):e34546. doi: 10.1371/journal.pone.0034546 (PMC3315571; doi:10.1371/journal.pone.0034546)
Supplement: Document S1 — Lack of correlation of miRNAs with platinum response. (DOC) [file pone.0034546.s005.doc]

**MicroRNAs fail to predict plantinum resistance in ovarian cancer**

**Background:** In this analysis, we generated a microRNA expression signature in an effort to predict the platinum resistance as a dichotomous variable. A variety of methods exist for binary classification and predictions, including, Support Vector Machine (SVM), Random Forest, Bayesian binary predictions etc. The challenging task with any such prediction method is finding an optimal subset of features from a high-dimensional dataset in addition to minimizing model prediction error rates. This challenge can be complicated by the following factors: the number of training samples is relatively small, the data is noisy, and the classes are highly un-balanced.

**Methodology:** Being aware of these challenges, we attempted binary prediction using several previously successful SVM methods. We limit the discussion here to show the recursive SVM (R-SVM) [1-4] algorithm performance. Essentially, R-SVM selects features that can best classify different subtypes of patients, and estimates the model prediction error, simultaneously. Current implementation of R-SVM evaluates the relative importance of features using the weighted difference of means of genes in the SVM decision function. Detailed methodology is available in <http://www.stanford.edu/group/wonglab/RSVMpage/R-SVM.html> and the original references [1-2].

TCGA batches 9 to 15 were used as training data and batches 17 to 22 as the unseen test data. Training data comprised 142 cases (50 Resistant and 92 Sensitive cases) and testing data comprised 143 cases (42 Resistant and 101 Sensitive cases). Leave-One-Out cross-validation (LOOCV) was performed on the training data.

**Results:** The result of the above methodology was an SVM model based on 15 microRNAs (Table 1) and LOOCV error equal to 32.4%. This trained SVM model was used to compute the performance on the un-seen test data (Figure 1). On the test data, the overall accuracy of the model was 65% and the area under the curve (AUC) resulting from Receiver Operating Characteristic curve (ROC) was 51.4% (Sensitivity=12%, Specificity=87%, Positive predictive value = 28% and Negative Predictive Value = 70% with ‘Positive’ Class being ‘Resistant’).

| hsa-let-7d | hsa-let-7e | hsa-let-7f | hsa-miR-107 | hsa-miR-10b |
| --- | --- | --- | --- | --- |
| hsa-miR-130a | hsa-miR-135b | hsa-miR-15b | hsa-miR-16 | hsa-miR-193b |
| hsa-miR-26b | hsa-miR-365 | hsa-miR-424 | hsa-miR-7 | hsa-miR-886-3p |

**Table 1:** Fifteen microRNAs resulting from the R-SVM LOOCV method.

**
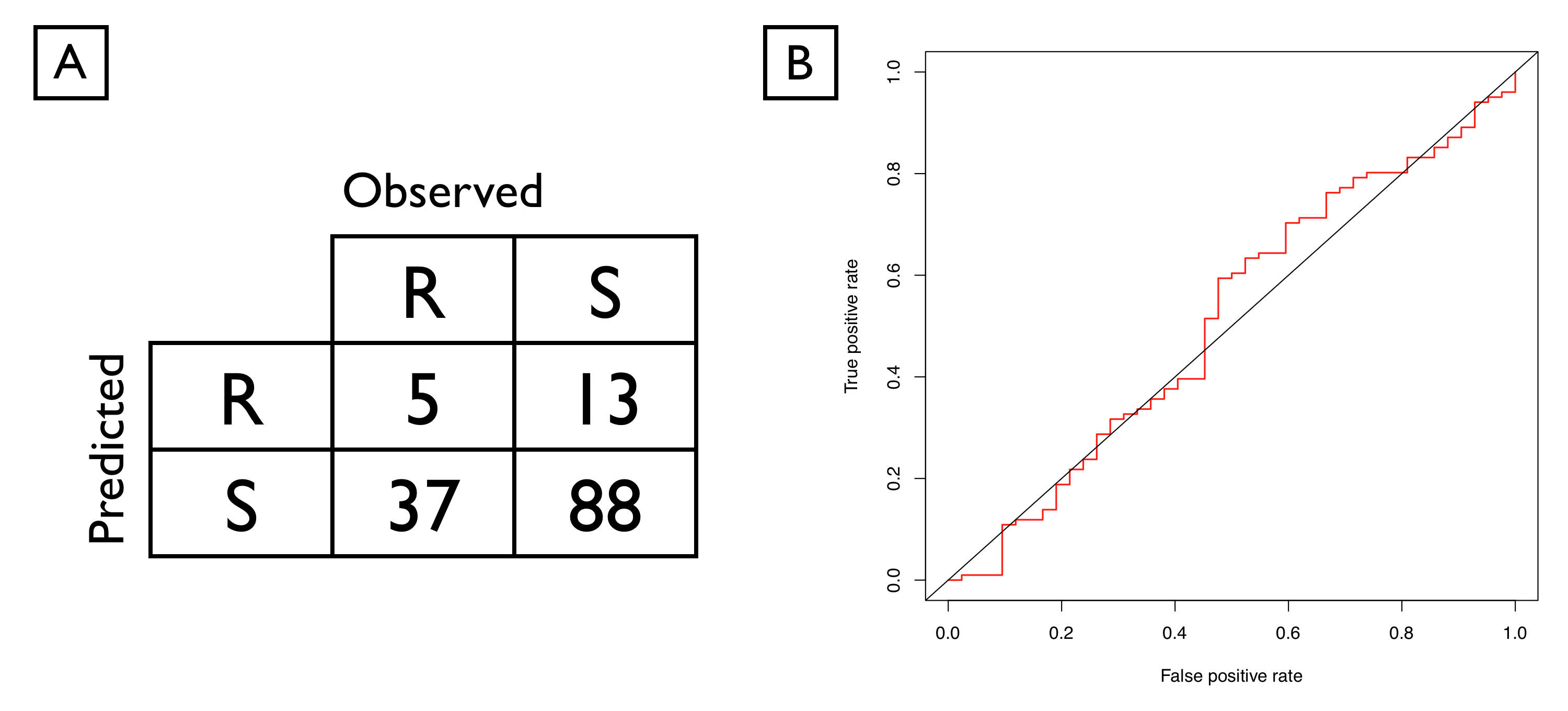
**

**Figure 1: Prediction performance of the test data:** (A) Contingency table with R=Resistant and S=Sensitive; and (B) ROC curve (AUC=51.4).

**Conclusions:** We conclude that microRNA expression is not a reliable predictor of platinum resistance as a dichotomous variable. Platinum-resistance in ovarian cancer is likely a complex and multifactorial process that is challenging to predict from pretreatment microRNA profiles obtained at the time of diagnosis.

**References:**

1) Xuegong Zhang, Wing H. Wong; *Recursive sample classification and gene selection based on SVM: method and software description*, Technical Report; Department of Biostatistics, Harvard School of Public Health, 2001.

2) Xuesong Lu, Xin Lu, *et. al*.; *Recursive SVM feature selection and sample classification for mass-spectrometry and microarray data*; BMC Bioinformatics, 2006, 7, 197-209.

3) Xuesong Lu, Xin Lu, *et. al.*; *Predicting features of breast cancer with gene expression patterns*; Breast Cancer Res Treat 2008, 108, 191-201.

4) Qian Shi, Lyndsay N. Harris, *et. al.*; *Declining Plasma Fibrinogen Alpha Fragment Identifies HER2-Positive Breast Cancer Patients and Reverts to Normal Levels after Surgery*; J. Proteasome Research, 2008, 5, 2947-2955.
