# Supplementary material for: Integrated Analyses of microRNAs Demonstrate Their Widespread Influence on Gene Expression in High-Grade Serous Ovarian Carcinoma
Source: PLoS One. 2012 Mar 29;7(3):e34546. doi: 10.1371/journal.pone.0034546 (PMC3315571; doi:10.1371/journal.pone.0034546)
Supplement: Document S2 — Functional gene classes associated with negatively correlation miR:gene pairs. (PDF) [file pone.0034546.s006.pdf]

# Clustering of miRNA:gene pairs

Anders Jacobsen

December 29, 2010

## Contents

1 Methods 1
1.1 cluster 1 . . . . . . . . . . 2
1.2 cluster 2 . . . . . . . . . . 4
1.3 cluster 3 . . . . . . . . . . 6
1.4 cluster 4 . . . . . . . . . . 8
1.5 cluster 5 . . . . . . . . . . 10
1.6 cluster 6 . . . . . . . . . . 12

## 1 Methods

We construct a matrix of correlation coefficients for all miRNA:gene pairs with a strong negative correlation (regression coefficient smaller than -7.0). This correlation matrix consists of 1760 genes and 35 miRNAs.

We cluster the miRNAs and genes separately based on the correlation matrix (hierarchical clustering, distance metric is pearson correlation coefficient, Ward's linkage). This way miRNAs are clustered in gene-space, grouping miRNAs when they are negatively correlated with same genes, and genes are clustered in miRNA-space, grouping genes when they are negatively correlated with the same miRNAs.

We cut the gene dendrogram to extract 6 gene clusters (colored in figure below). In the following sections each of these gene clusters are extracted and shown in more detail. Furthermore, the genes in each cluster are analyzed for functional enrichment in GO Molecular Functions, GO Biological Process and KEGG pathways (relative to gene universe consisting of all genes on the array).

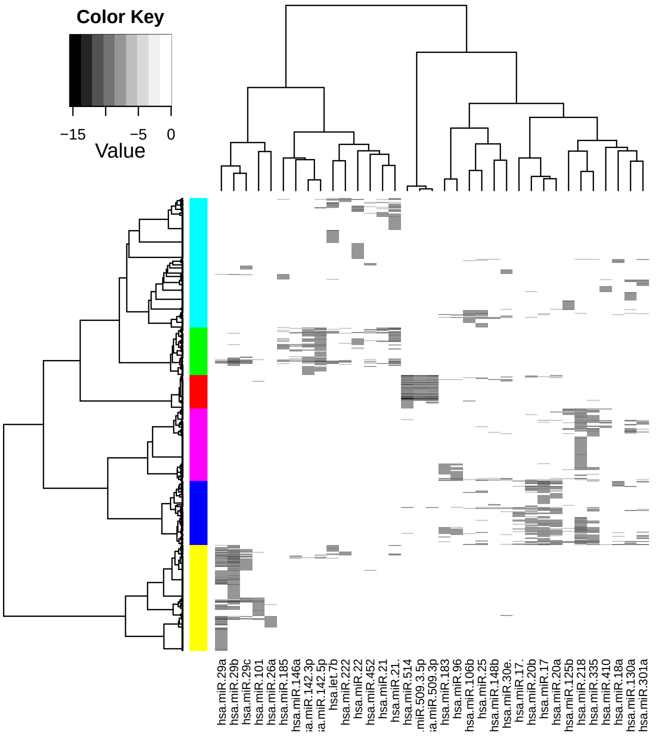
Color Key
−15
−5
0
Value
hsa.miR.29a
hsa.miR.29b
hsa.miR.29c
hsa.miR.101
hsa.miR.26a
hsa.miR.185
hsa.miR.146a
a.miR.142.3p
a.miR.142.5p
hsa.let.7b
hsa.miR.222
hsa.miR.22
hsa.miR.452
hsa.miR.21
hsa.miR.21.
hsa.miR.514
miR.509.3.5p
a.miR.509.3p
hsa.miR.183
hsa.miR.96
hsa.miR.106b
hsa.miR.25
hsa.miR.148b
hsa.miR.30e.
hsa.miR.17.
hsa.miR.20b
hsa.miR.17
hsa.miR.20a
hsa.miR.125b
hsa.miR.218
hsa.miR.335
hsa.miR.410
hsa.miR.18a
hsa.miR.130a
hsa.miR.301a

## 1.1 cluster 1

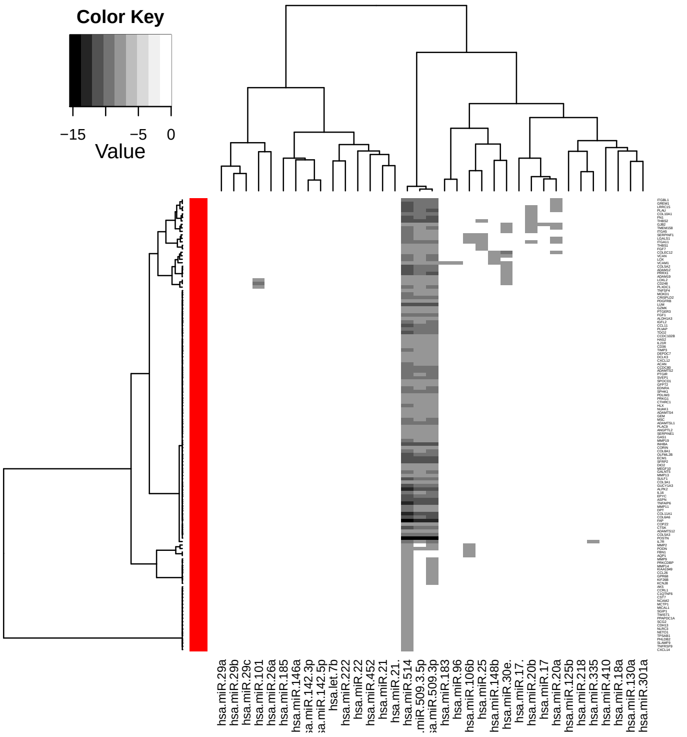
Color Key
−15
−5
0
Value
hsa.miR.29a
hsa.miR.29b
hsa.miR.29c
hsa.miR.101
hsa.miR.26a
hsa.miR.185
hsa.miR.146a
a.miR.142.3p
a.miR.142.5p
hsa.let.7b
hsa.miR.222
hsa.miR.22
hsa.miR.452
hsa.miR.21
hsa.miR.21.
hsa.miR.514
miR.509.3.5p
a.miR.509.3p
hsa.miR.183
hsa.miR.96
hsa.miR.106b
hsa.miR.25
hsa.miR.148b
hsa.miR.30e.
hsa.miR.17.
hsa.miR.20b
hsa.miR.17
hsa.miR.20a
hsa.miR.125b
hsa.miR.218
hsa.miR.335
hsa.miR.410
hsa.miR.18a
hsa.miR.130a
hsa.miR.301a

| | GOBPID | Pvalue | Count | Size | Term |
|---|---|---|---|---|---|
| 1 | GO:0030199 | 1.15e-09 | 7 | 24 | collagen fibril organization |
| 2 | GO:0007155 | 4.24e-09 | 22 | 596 | cell adhesion |
| 3 | GO:0044259 | 2.06e-08 | 7 | 35 | multicellular organismal macromolecule metabolic process |
| 4 | GO:0030574 | 2.16e-08 | 6 | 21 | collagen catabolic process |
| 5 | GO:0040011 | 7.27e-08 | 17 | 406 | locomotion |

Table 1: Cluster 1 : GO Biological Process

| | GOMFID | Pvalue | Count | Size | Term |
|---|---|---|---|---|---|
| 1 | GO:0004222 | 1.03e-10 | 12 | 101 | metalloendopeptidase activity |
| 2 | GO:0030247 | 1.27e-09 | 12 | 125 | polysaccharide binding |
| 3 | GO:0070011 | 4.14e-08 | 19 | 471 | peptidase activity, acting on L-amino acid peptides |
| 4 | GO:0005201 | 3.24e-07 | 8 | 74 | extracellular matrix structural constituent |
| 5 | GO:0008201 | 8.69e-07 | 8 | 84 | heparin binding |

Table 2: Cluster 1 : GO Molecular Function

| | KEGGID | Pvalue | Count | Size | Term |
|---|---|---|---|---|---|
| 1 | 04512 | 1.68e-10 | 11 | 82 | ECM-receptor interaction |
| 2 | 04510 | 1.12e-06 | 11 | 188 | Focal adhesion |
| 3 | 04060 | 8.51e-05 | 10 | 244 | Cytokine-cytokine receptor interaction |
| 4 | 05219 | 6.13e-03 | 3 | 39 | Bladder cancer |

Table 3: Cluster 1 : KEGG Pathways

## 1.2 cluster 2

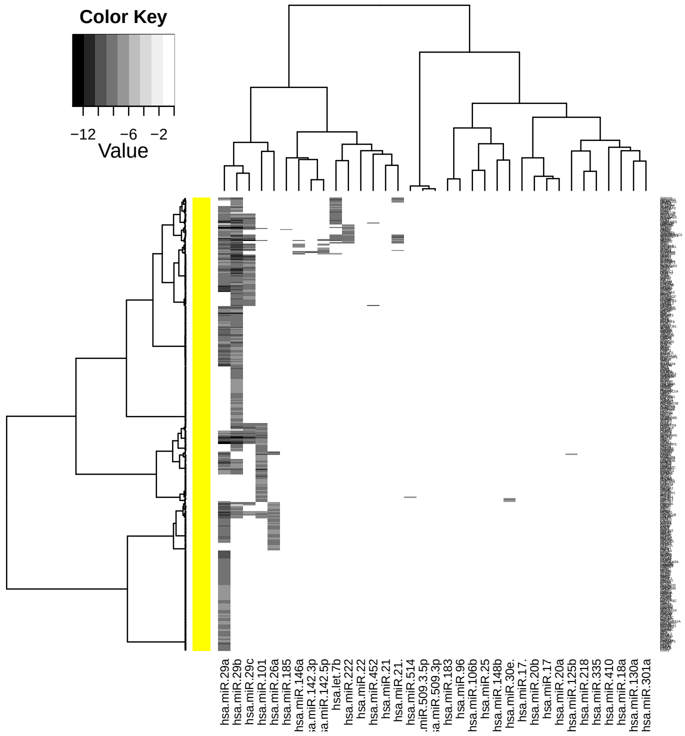


| | GOBPID | Pvalue | Count | Size | Term |
|---|---|---|---|---|---|
| 1 | GO:0048285 | 5.70e-39 | 57 | 222 | organelle fission |
| 2 | GO:0051301 | 1.83e-36 | 53 | 209 | cell division |
| 3 | GO:0000279 | 5.61e-33 | 56 | 280 | M phase |
| 4 | GO:0007067 | 3.79e-27 | 37 | 137 | mitosis |
| 5 | GO:0000278 | 5.98e-27 | 49 | 275 | mitotic cell cycle |

Table 4: Cluster 2 : GO Biological Process

| | GOMFID | Pvalue | Count | Size | Term |
|---|---|---|---|---|---|
| 1 | GO:0005515 | 1.83e-07 | 231 | 6712 | protein binding |
| 2 | GO:0032555 | 1.60e-05 | 73 | 1603 | purine ribonucleotide binding |
| 3 | GO:0005524 | 1.75e-05 | 62 | 1294 | ATP binding |
| 4 | GO:0000166 | 4.51e-05 | 82 | 1920 | nucleotide binding |
| 5 | GO:0017111 | 8.71e-05 | 35 | 629 | nucleoside-triphosphatase activity |

Table 5: Cluster 2 : GO Molecular Function

|  | KEGGID | Pvalue | Count | Size | Term |
|---|---|---|---|---|---|
| 1 | 04110 | 1.44e-17 | 27 | 120 | Cell cycle |
| 2 | 04115 | 9.29e-05 | 9 | 65 | p53 signaling pathway |
| 3 | 03410 | 2.66e-04 | 6 | 32 | Base excision repair |
| 4 | 05130 | 2.46e-03 | 6 | 48 | Pathogenic Escherichia coli infection - EHEC |
| 5 | 03440 | 7.19e-03 | 4 | 27 | Homologous recombination |

Table 6: Cluster 2 : KEGG Pathways

## 1.3 cluster 3

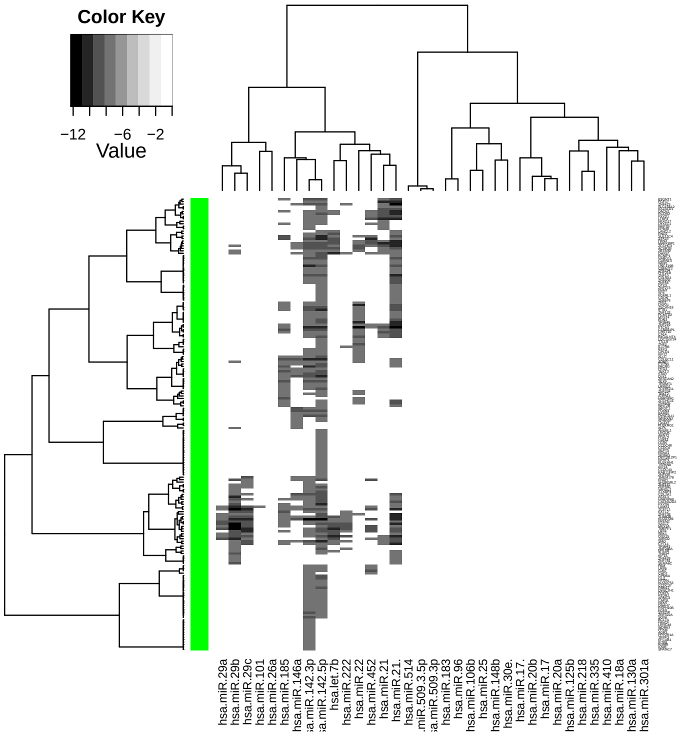


| | GOBPID | Pvalue | Count | Size | Term |
|---|---|---|---|---|---|
| 1 | GO:0051171 | 1.41e-06 | 49 | 2367 | regulation of nitrogen compound metabolic process |
| 2 | GO:0080090 | 1.56e-06 | 54 | 2748 | regulation of primary metabolic process |
| 3 | GO:0010467 | 4.09e-06 | 58 | 3144 | gene expression |
| 4 | GO:0009889 | 7.84e-06 | 41 | 2042 | regulation of biosynthetic process |
| 5 | GO:0034961 | 1.55e-05 | 52 | 2800 | cellular biopolymer biosynthetic process |

Table 7: Cluster 3 : GO Biological Process

| | GOMFID | Pvalue | Count | Size | Term |
|---|---|---|---|---|---|
| 1 | GO:0008270 | 7.55e-06 | 45 | 1954 | zinc ion binding |
| 2 | GO:0003700 | 1.11e-03 | 21 | 839 | transcription factor activity |
| 3 | GO:0003777 | 1.17e-03 | 5 | 66 | microtubule motor activity |
| 4 | GO:0046872 | 2.03e-03 | 60 | 3578 | metal ion binding |
| 5 | GO:0030020 | 2.09e-03 | 2 | 6 | extracellular matrix structural constituent conferring tensile strengt |

Table 8: Cluster 3 : GO Molecular Function

| | KEGGID | Pvalue | Count | Size | Term |
|---|---|---|---|---|---|
| 1 | 05217 | 5.59e-05 | 5 | 55 | Basal cell carcinoma |
| 2 | 05200 | 9.48e-05 | 10 | 315 | Pathways in cancer |
| 3 | 04340 | 7.91e-04 | 4 | 55 | Hedgehog signaling pathway |
| 4 | 04512 | 3.49e-03 | 4 | 82 | ECM-receptor interaction |
| 5 | 04310 | 4.69e-03 | 5 | 144 | Wnt signaling pathway |

Table 9: Cluster 3 : KEGG Pathways

## 1.4 cluster 4

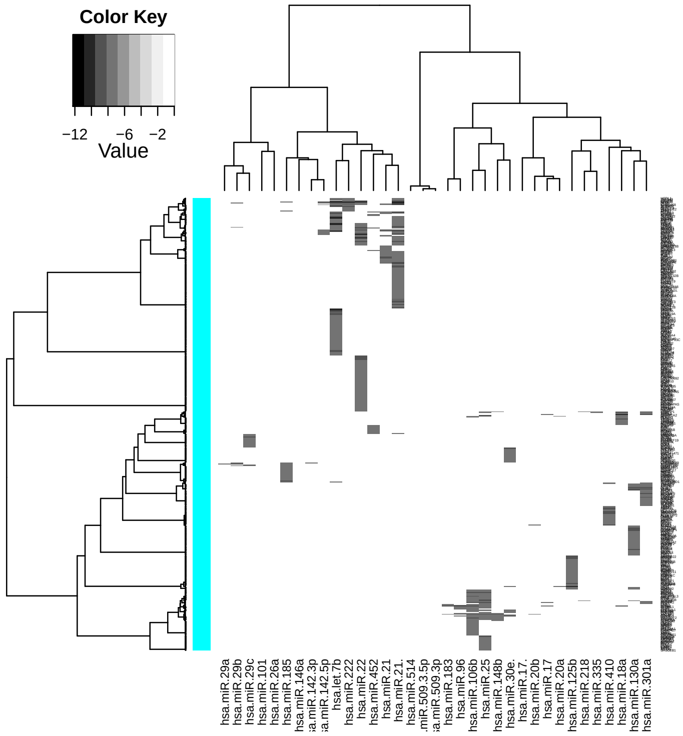


| | GOBPID | Pvalue | Count | Size | Term |
|---|---|---|---|---|---|
| 1 | GO:0043284 | 2.52e-04 | 123 | 2810 | biopolymer biosynthetic process |
| 2 | GO:0010556 | 3.16e-04 | 107 | 2388 | regulation of macromolecule biosynthetic process |
| 3 | GO:0048146 | 5.01e-04 | 5 | 21 | positive regulation of fibroblast proliferation |
| 4 | GO:0034645 | 7.11e-04 | 127 | 2992 | cellular macromolecule biosynthetic process |
| 5 | GO:0006350 | 7.12e-04 | 101 | 2277 | transcription |

Table 10: Cluster 4 : GO Biological Process

| | GOMFID | Pvalue | Count | Size | Term |
|---|---|---|---|---|---|
| 1 | GO:0008270 | 3.58e-09 | 109 | 1954 | zinc ion binding |
| 2 | GO:0043167 | 1.01e-06 | 164 | 3660 | ion binding |
| 3 | GO:0046872 | 1.05e-06 | 161 | 3578 | metal ion binding |
| 4 | GO:0005488 | 3.62e-04 | 176 | 5954 | binding |
| 5 | GO:0003677 | 3.76e-04 | 91 | 1996 | DNA binding |

Table 11: Cluster 4 : GO Molecular Function

|   | KEGGID | Pvalue | Count | Size | Term |
|---|---|---|---|---|---|
| 1 | 04115 | 4.30e-04 | 8 | 65 | p53 signaling pathway |
| 2 | 00230 | 7.50e-03 | 10 | 145 | Purine metabolism |
| 3 | 05214 | 7.66e-03 | 6 | 62 | Glioma |
| 4 | 04530 | 8.42e-03 | 9 | 125 | Tight junction |

Table 12: Cluster 4 : KEGG Pathways

## 1.5 cluster 5

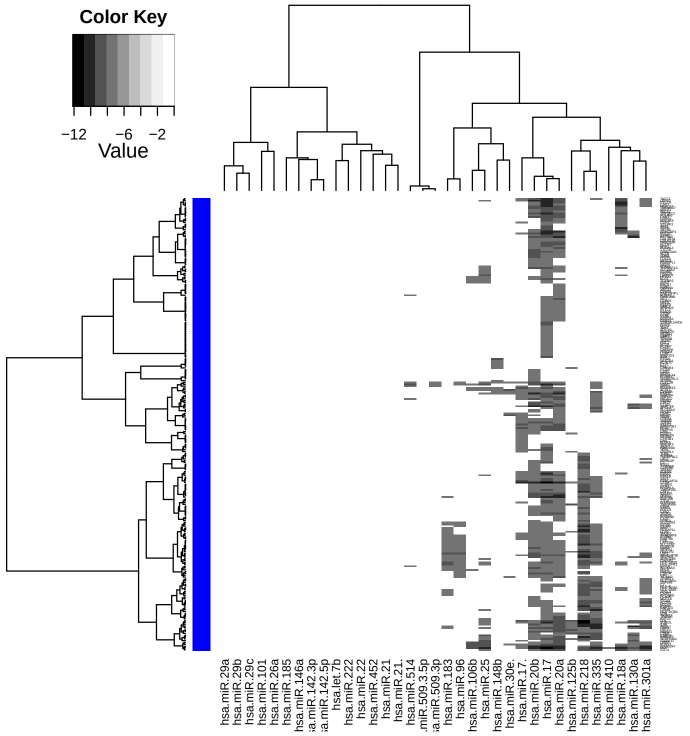
Color Key
−12
−6
−2
Value
hsa.miR.29a
hsa.miR.29b
hsa.miR.29c
hsa.miR.101
hsa.miR.26a
hsa.miR.185
hsa.miR.146a
a.miR.142.3p
a.miR.142.5p
hsa.let.7b
hsa.miR.222
hsa.miR.22
hsa.miR.452
hsa.miR.21
hsa.miR.21.
hsa.miR.514
.miR.509.3.5p
a.miR.509.3p
hsa.miR.183
hsa.miR.96
hsa.miR.106b
hsa.miR.25
hsa.miR.148b
hsa.miR.30e.
hsa.miR.17.
hsa.miR.20b
hsa.miR.17
hsa.miR.20a
hsa.miR.125b
hsa.miR.218
hsa.miR.335
hsa.miR.410
hsa.miR.18a
hsa.miR.130a
hsa.miR.301a

| | GOBPID | Pvalue | Count | Size | Term |
|---|---|---|---|---|---|
| 1 | GO:0006955 | 2.14e-09 | 23 | 335 | immune response |
| 2 | GO:0002504 | 1.42e-07 | 6 | 16 | antigen processing and presentation of peptide or polysaccharide anti |
| 3 | GO:0002274 | 9.66e-07 | 8 | 47 | myeloid leukocyte activation |
| 4 | GO:0006954 | 1.99e-06 | 18 | 295 | inflammatory response |
| 5 | GO:0010627 | 6.31e-06 | 14 | 199 | regulation of protein kinase cascade |

Table 13: Cluster 5 : GO Biological Process

| | GOMFID | Pvalue | Count | Size | Term |
|---|---|---|---|---|---|
| 1 | GO:0032395 | 4.86e-06 | 4 | 8 | MHC class II receptor activity |
| 2 | GO:0019955 | 8.90e-05 | 8 | 87 | cytokine binding |
| 3 | GO:0019864 | 2.36e-04 | 3 | 8 | IgG binding |
| 4 | GO:0051015 | 2.95e-04 | 5 | 36 | actin filament binding |
| 5 | GO:0005057 | 9.63e-04 | 9 | 152 | receptor signaling protein activity |

Table 14: Cluster 5 : GO Molecular Function

| | KEGGID | Pvalue | Count | Size | Term |
|---|---|---|---|---|---|
| 1 | 05330 | 4.40e-07 | 8 | 34 | Allograft rejection |
| 2 | 05332 | 8.85e-07 | 8 | 37 | Graft-versus-host disease |
| 3 | 05322 | 1.10e-06 | 13 | 117 | Systemic lupus erythematosus |
| 4 | 04940 | 2.04e-06 | 8 | 41 | Type I diabetes mellitus |
| 5 | 05320 | 7.10e-06 | 8 | 48 | Autoimmune thyroid disease |

Table 15: Cluster 5 : KEGG Pathways

## 1.6 cluster 6

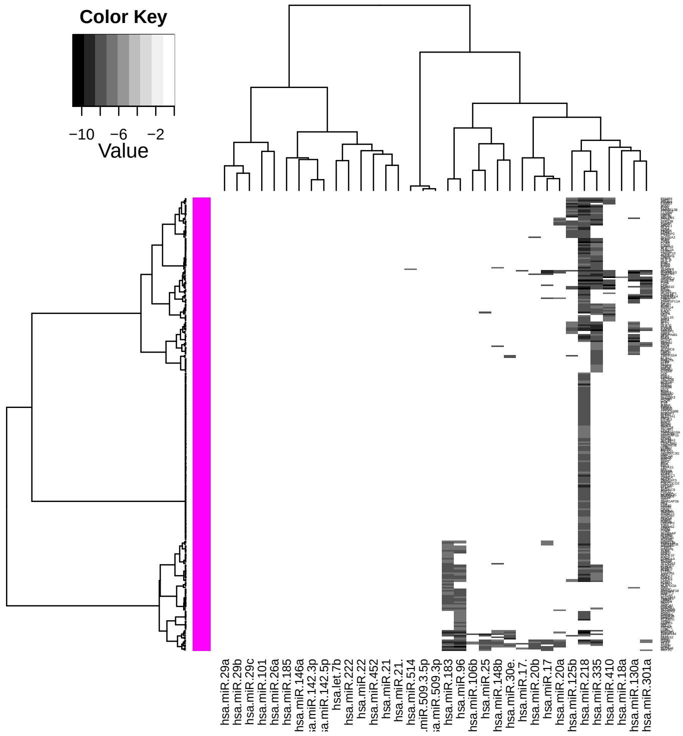


| | GOBPID | Pvalue | Count | Size | Term |
|---|---|---|---|---|---|
| 1 | GO:0006955 | 8.59e-29 | 47 | 369 | immune response |
| 2 | GO:0006954 | 3.80e-14 | 26 | 220 | inflammatory response |
| 3 | GO:0009605 | 2.75e-11 | 36 | 562 | response to external stimulus |
| 4 | GO:0045087 | 6.60e-11 | 16 | 105 | innate immune response |
| 5 | GO:0002474 | 2.33e-10 | 7 | 11 | antigen processing and presentation of peptide antigen via MHC clas |

Table 16: Cluster 6 : GO Biological Process

| | GOMFID | Pvalue | Count | Size | Term |
|---|---|---|---|---|---|
| 1 | GO:0004872 | 8.69e-07 | 53 | 1449 | receptor activity |
| 2 | GO:0032393 | 2.66e-06 | 5 | 13 | MHC class I receptor activity |
| 3 | GO:0015026 | 1.64e-05 | 5 | 18 | coreceptor activity |
| 4 | GO:0042803 | 3.13e-05 | 16 | 257 | protein homodimerization activity |
| 5 | GO:0001875 | 6.53e-05 | 3 | 5 | lipopolysaccharide receptor activity |

Table 17: Cluster 6 : GO Molecular Function

| | KEGGID | Pvalue | Count | Size | Term |
|---|---|---|---|---|---|
| 1 | 04612 | 5.57e-11 | 16 | 79 | Antigen processing and presentation |
| 2 | 04620 | 1.04e-08 | 15 | 96 | Toll-like receptor signaling pathway |
| 3 | 04514 | 4.84e-08 | 16 | 123 | Cell adhesion molecules (CAMs) |
| 4 | 05330 | 1.37e-06 | 8 | 34 | Allograft rejection |
| 5 | 05332 | 2.73e-06 | 8 | 37 | Graft-versus-host disease |

Table 18: Cluster 6 : KEGG Pathways
