# Supplementary figures and images for: Integrated Analyses of microRNAs Demonstrate Their Widespread Influence on Gene Expression in High-Grade Serous Ovarian Carcinoma
Source: PLoS One. 2012 Mar 29;7(3):e34546. doi: 10.1371/journal.pone.0034546 (PMC3315571; doi:10.1371/journal.pone.0034546)

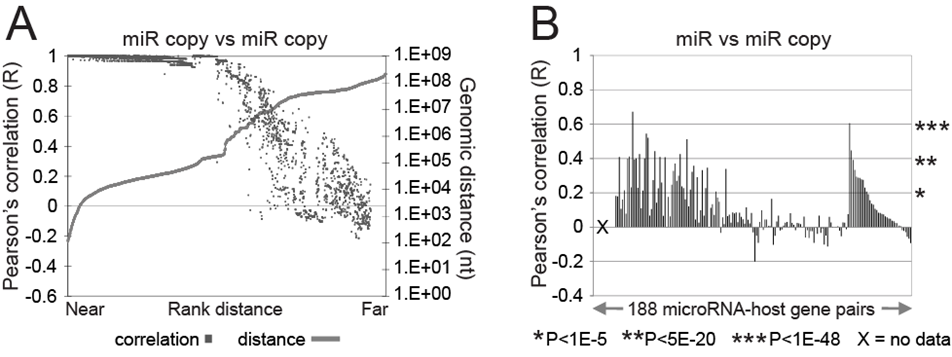

Supplement: Figure S1 — The observed patterns of miRNAs (miRs) being frequently coexpressed with neighboring miRNAs and host genes are not solely due to copy number alterations. (A) As in main Figure 1A, but with the correlations between miRNA copy levels (SNP 1 M dataset, collapsed into miRNAs). (B) For purposes of comparison with the results of main Figure 1B, for each of 188 miRNA-host gene pairs (same orientation), the correlation was computed between miRNA expression and host gene copy (part D, using MSKCC 1 M CGH dataset, averaged by gene); ordering of mRNA-host gene pairs is the same between main Figure 1B and Supplemental Figure 1B; “X”, no corresponding mRNA or gene copy data. (PNG) [file pone.0034546.s007.png]

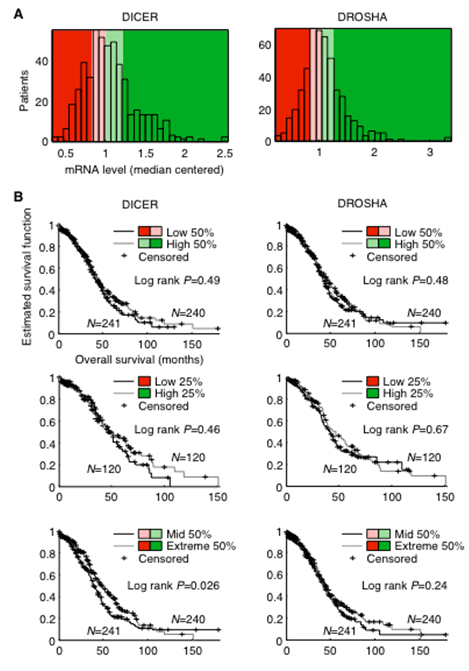

Supplement: Figure S2 — Lack of association between Dicer and Drosha levels with overall survival in the TCGA cohort. A previous study of 111 invasive epithelial ovarian cancer samples found that the distribution of Dicer mRNA levels was bimodal, and that both Dicer and Drosha levels were positively associated with overall survival (Merritt et al, 2008). (A) Dicer mRNA was not bimodal in TCGA. (B) The data did not support a significant difference in overall survival between patients with low and high Drosha or Dicer levels. This was the case when comparing the top and bottom 50% of patients as well as the lower and upper quartiles (P>0.45, logrank test). A marginal improvement in overall survival was seen in patients where Dicer levels were in the extreme upper and lower quartiles (P = 0.026, logrank test), although median survival for uncensored patients was not improved in this case. Likewise, no association was revealed by Cox proportional hazards regression (P = 0.45 and P = 0.53 for Dicer and Drosha, respectively). (PNG) [file pone.0034546.s008.png]

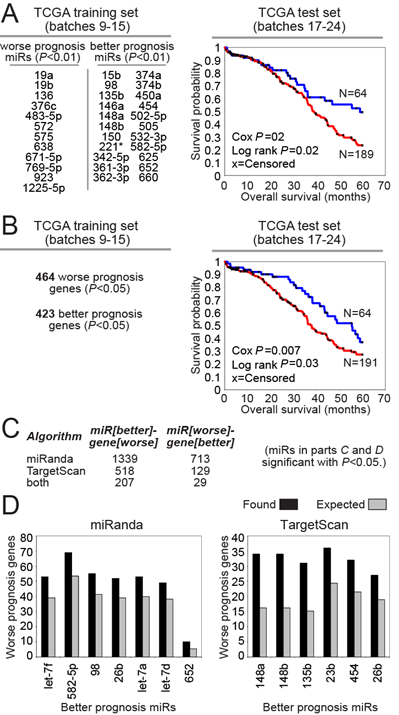

Supplement: Figure S3 — miRNAs and associated genes correlated with patient survival in ovarian cancer. (A) Using a training dataset of TCGA microRNA expression profiles, a prognostic gene signature was defined (left panel) and then applied to a test dataset (right panel), each tumor being assigned a score measuring how well the tumor's expression patterns reflected those of the signature pattern. Kaplan-Meier analysis (log-rank tests) compares time to death for ovarian cancer patients showing higher risk (prognostic score>0) versus lower risk (prognositc score<0). Univariate Cox test treats the prognostic score as a continuous variable. (B) As for part A, but using gene expression profiles to define survival gene correlates. (C) Numbers of predicted miRNA-mRNA functional pairs for each algorithm and intersection of algorithms based on anti-correlated expression in ovarian cancer. (D) Top enriched miRNA targeting associations (one-sided Fisher's exact of P<0.05) for the genes correlated with better prognosis, for the given algorithm. (PNG) [file pone.0034546.s009.png]

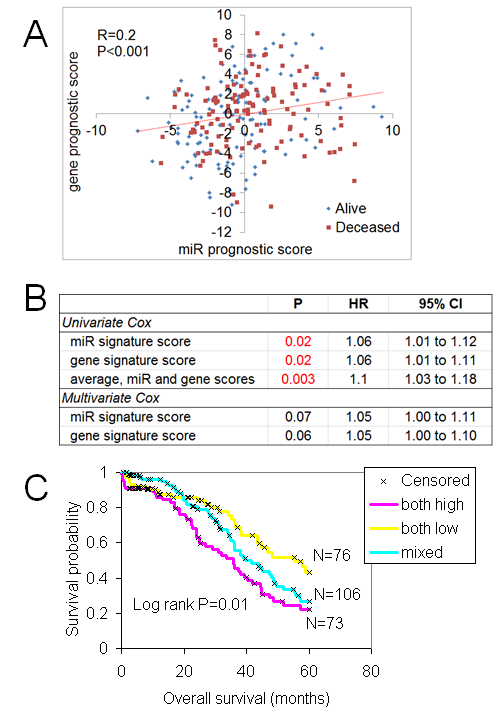

Supplement: Figure S4 — Comparison of prognostic signature scores derived from miRNA versus gene expression. Previously (TCGA consortium, 2011), a gene expression signature of prognosis was derived and applied to the same validation dataset used to validate our miRNA prognostic signature. (A) Scatterplot comparing miR and gene prognostic scores in the validation cohort (N = 253). R-value and P-value by Pearson's. (B) Cox survival analysis of miR and gene prognostic signature scores (evaluated as a continuous variable). The scores were evaluated individually by univariate Cox, as well as an averaging of the two scores. By multivariate Cox, the scores were evaluated in the same model. (C) Kaplan-Meier analysis evaluating survival time for three groups of patients: score>0 for both miR and gene (pink), score<0 for both miR and gene (yellow), and all others (blue). (PNG) [file pone.0034546.s010.png]

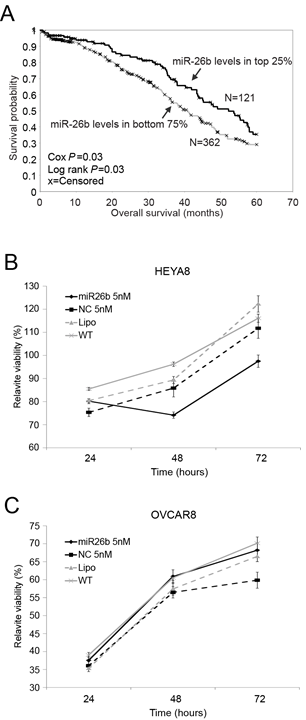

Supplement: Figure S5 — miR-26b, associated with longer survival in ovarian cancer patients, impacts cell viability in vitro in HEYA8 cell line but not in OVCAR8. (A) Kaplan-Meier analysis evaluating survival time for patient with higher versus lower levels of miR-26b. Univariate Cox test evaluates miR-26b expression as a continuous variable; Log rank test compares the top 25% of expressors with the rest of the patients. (B) MTS assays demonstrating the effect of miR-26b overexpression on proliferation of HEYA8 cells (Lipo, lipofectamine-treated alone, no miRNA; two-sided t-test P< = 0.01, miR-26b vs each of three control groups at both 48 h and 72 h). (C) No effect was observed for miR-26b on proliferation of OVCAR-8 cells. Over-expression and MTS experiments for miR-26b were carried out in the same manner as for the miR-29a experiments of Figure 6D. (PNG) [file pone.0034546.s011.png]

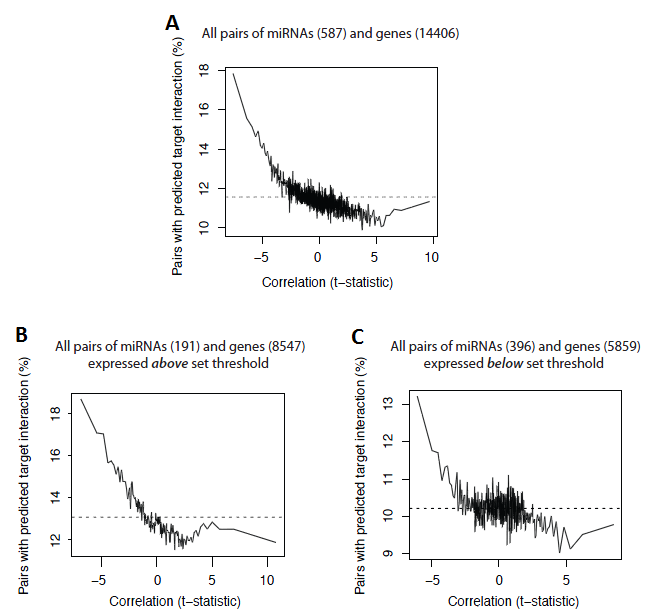

Supplement: Figure S6 — MiRNAs and their predicted gene targets tend to be anti-correlated within ovarian tumors. Similar to main Figure 4A, except here all miRNAs and genes represented in the dataset are considered (A), in addition to the top most expressed miRNAs and genes (B), from Figure 4A, as well as the remaining miRNAs and genes expressed below the set threshold (C). miRNA:gene pairs are subdivided into bins of 10,000 each. Miranda predictions and Regression model results were used. (PNG) [file pone.0034546.s012.png]

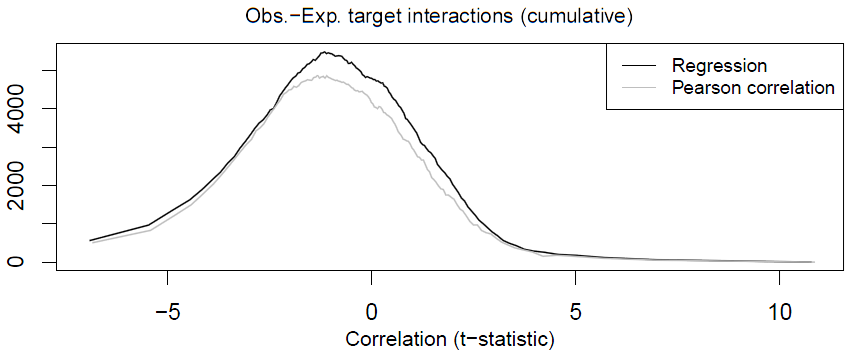

Supplement: Figure S7 — MiRNAs and their predicted gene targets tend to be anti-correlated within ovarian tumors, with the regression model's negatively correlated pairs showing slightly greater target enrichment as compared to the Pearson's model. Scatter plot showing cumulative mean correlation and fraction of predicted target interactions (miRanda-mirSVR score>0.1), using bins of 10,000 miRNA:gene pairs (total number of pairs represented: 191 miRNAs X 8547 genes). (Similar to main Figure 4A, except number of interactions is cumulated from low to high correlation.) (PNG) [file pone.0034546.s013.png]

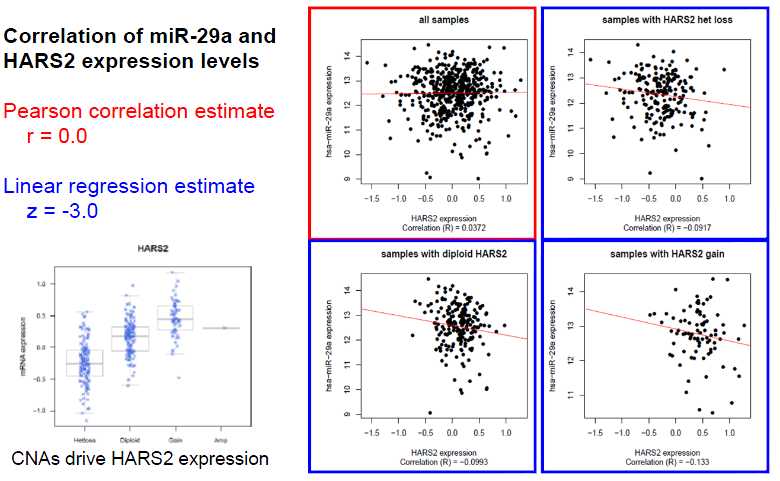

Supplement: Figure S8 — miR-29a:HARS2 as an example demonstrating correction of CNA bias in miRNA:gene expression correlation. Correlations were computed using both Pearson's correlation (scatter plot outlined in red) and a simple linear regression model to account for ‘noise’ due to DNA copy alterations (scatter plots outlined in blue). (PNG) [file pone.0034546.s014.png]

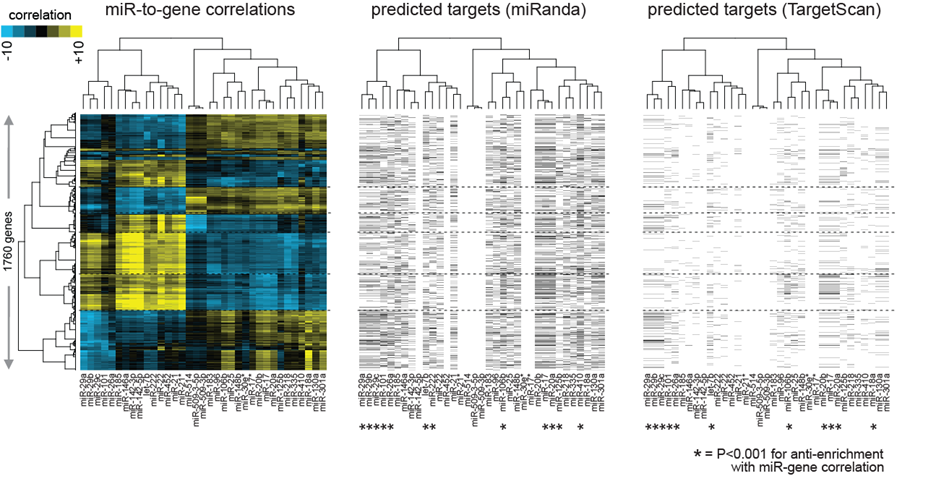

Supplement: Figure S9 — Within ovarian tumors, several specific miRNAs tend to be anti-correlated with their predicted gene targets. Left panel shows hierarchical clustering matrix (with pearson correlation coefficient as distance metric,Ward's Linkage) of correlation coefficients for all miRNA:gene pairs (yellow = positive correlation; blue = negative correlation). The two right panels show the corresponding predicted targeting interaction (both PicTar and TargetScan algorithms) for the miRNAs/genes (same gene ordering). *, significant anti-enrichment (P<0.001, Spearman's rank, one-sided) for predicted targets within miRNA-to-gene correlations. (PNG) [file pone.0034546.s015.png]

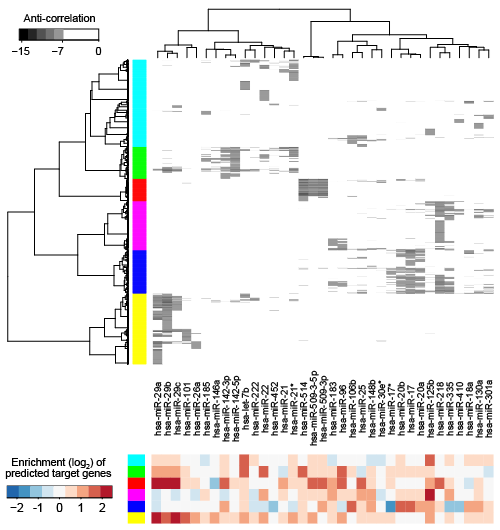

Supplement: Figure S10 — For each miRNA in the miRNA:gene correlation matrix (from Figure 4 ), enrichment of predicted target genes within each of the six different gene clusters. Enrichment (bottom panel) is given by the fraction of predicted target genes in a given cluster (miRanda miRSVR score<−1.0), divided by the background expected ratio (overall fraction of miRNA target genes among all genes measured on array and with predicted target sites of any human miRNA). (PNG) [file pone.0034546.s016.png]

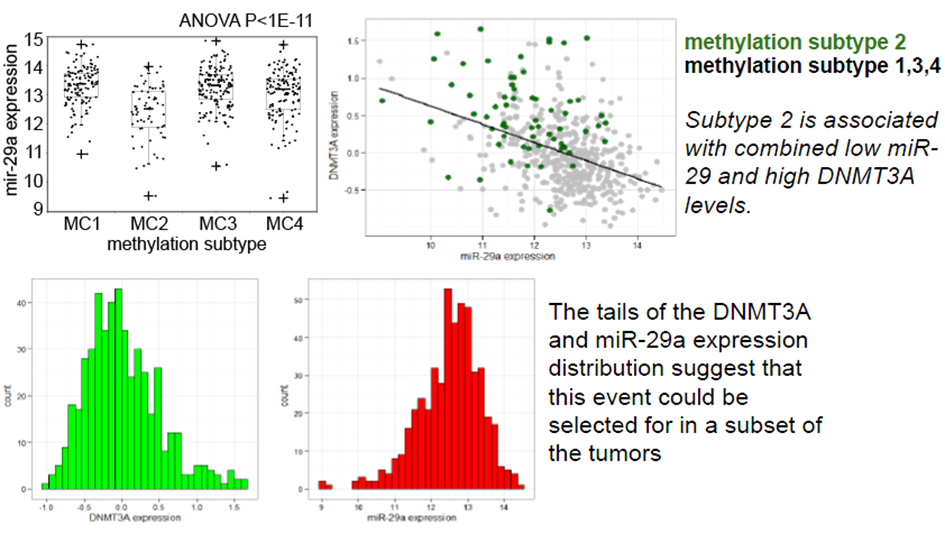

Supplement: Figure S11 — Methylation subtype 2 is associated with combined low miR-29 and high DNMT3A levels. (PNG) [file pone.0034546.s017.png]

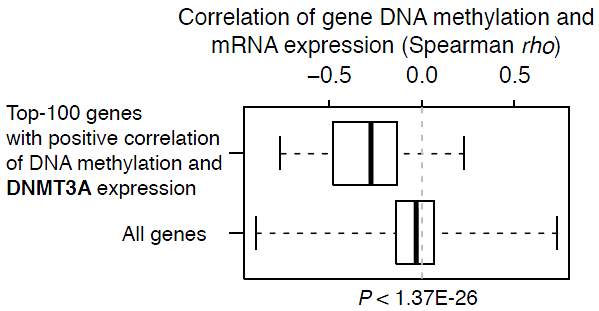

Supplement: Figure S12 — Possible targets of DNMT3A methylation in the ovarian tumors (having DNA methylation levels positively correlated with DNMT3A expression level) show a significantly higher proportion of genes with mRNA expression levels strongly affected by DNA methylation (P<1.4Ee-26, Wilcoxon Rank-sum). (PNG) [file pone.0034546.s018.png]
