# Supplementary material for: Integrated Analyses of microRNAs Demonstrate Their Widespread Influence on Gene Expression in High-Grade Serous Ovarian Carcinoma
Source: PLoS One. 2012 Mar 29;7(3):e34546. doi: 10.1371/journal.pone.0034546 (PMC3315571; doi:10.1371/journal.pone.0034546)
Supplement: Table S2 — PCR primer sequences. (DOCX) [file pone.0034546.s020.docx]

| Supplemental Table 1. PCR primer sequences. | |
| --- | --- |
| Target gene: | Primer sequence |
| TIMELESS | LEFT 5’-TTGCAGAACTGGAGGTGTTG-3’ |
| TIMELESS | RIGHT AGGTTGTGAAGGCCTTTGTG |
| CDC6 | LEFT 5’-GCTGTTGAACTTCCCACCTT-3’ |
| CDC6 | RIGHT 5’-TCTCCTGCAAACATCCAGTG-3’ |
| DNMT3B | LEFT 5’-AGATCAAGCTCGCGACTCTC-3’ |
| DNMT3B | RIGHT 5’-GACAGCTGGGCTTTCTGAAC-3’ |
| DNMT3A | LEFT 5’-GGACAAGAATGCCACCAAAG-3’ |
| DNMT3A | RIGHT 5’-CCACTGAGAATTTGCCGTCT-3’ |
| MYBL2 | LEFT 5’-CACCTGGAGGAGGACTTGAA-3’ |
| MYBL2 | RIGHT 5’-CCACAATGTCAAGAGCCAGA-3’ |
| CBX1 | LEFT 5’-GGAGCGGATTATTGGAGCTA-3’ |
| CBX1 | RIGHT 5’-ATCCTCCGAGGGGTAGGAAT-3’ |
